# Large-effect pleiotropic or closely linked QTL segregate within and across ten US cattle breeds

**DOI:** 10.1186/1471-2164-15-442

**Published:** 2014-06-06

**Authors:** Mahdi Saatchi, Robert D Schnabel, Jeremy F Taylor, Dorian J Garrick

**Affiliations:** Department of Animal Science, Iowa State University, Ames, 50011 USA; Division of Animal Sciences, University of Missouri, Columbia, 65211 USA; Institute of Veterinary, Animal and Biomedical Sciences, Massey University, Palmerston North, New Zealand

**Keywords:** Candidate gene, Cattle, GWAS, Pleiotropy, QTL, SNP

## Abstract

**Background:**

The availability of high-density SNP assays including the BovineSNP50 (50 K) enables the identification of novel quantitative trait loci (QTL) and improvement of the resolution of the locations of previously mapped QTL. We performed a series of genome-wide association studies (GWAS) using 50 K genotypes scored in 18,274 animals from 10 US beef cattle breeds with observations for twelve body weights, calving ease and carcass traits.

**Results:**

A total of 159 large-effects QTL (defined as 1-Mb genome windows explaining more than 1% of additive genetic variance) were identified. In general, more QTL were identified in analyses with bigger sample sizes. Four large-effect pleiotropic or closely linked QTLs located on BTA6 at 37–42 Mb (primarily at 38 Mb), on BTA7 at 93 Mb, on BTA14 at 23–26 Mb (primarily at 25 Mb) and on BTA20 at 4 Mb were identified in more than one breed. Several breed-specific large-effect pleiotropic or closely linked QTL were also identified. Some identified QTL regions harbor genes known to have large effects on a variety of traits in cattle such as *PLAG1* and *MSTN* and others harbor promising candidate genes including *NCAPG*, *ARRDC3*, *ERGIC1*, *SH3PXD2B*, *HMGA2*, *MSRB3*, *LEMD3*, *TIGAR*, *SEPT7*, and *KIRREL3*. Gene ontology analysis revealed that genes involved in ossification and in adipose tissue development were over-represented in the identified pleiotropic QTL. Also, the MAPK signaling pathway was identified as a common pathway affected by the genes located near the pleiotropic QTL.

**Conclusions:**

This largest GWAS ever performed in beef cattle, led us to discover several novel across-breed and breed-specific large-effect pleiotropic QTL that cumulatively account for a significant percentage of additive genetic variance (e.g. more than a third of additive genetic variance of birth and mature weights; and calving ease direct in Hereford). These results will improve our understanding of the biology of growth and body composition in cattle.

**Electronic supplementary material:**

The online version of this article (doi:10.1186/1471-2164-15-442) contains supplementary material, which is available to authorized users.

## Background

Developments in molecular biology and statistical methodologies have made it possible for the dissection of genetic variation and the localization of economically important quantitative trait loci (QTL) in farm animals [1]. Many QTL associated with economically important traits in cattle have been reported and deposited in the AnimalQTLdb (http://www.animalgenome.org). However, not all of the genetic variation in these traits has been captured because of inadequate sample size and the low density of markers historically used in QTL mapping studies. Consequently, confidence intervals obtained for most reported QTL are too large (regions extending over several megabases (Mb)) to allow the identification of appropriate positional candidate gene(s) for the majority of QTL. Recent availability of high-density SNP assays that span the genome at high resolution such as the BovineSNP50 BeadChip [2] now enables the identification of novel QTL and improvement of the resolution of the location of previously mapped QTL.

Genome-wide association studies (GWAS) sometimes identify loci that are not responsible for variation (*i.e.*, false positives) because [3]: 1) stochastic noise can generate false associations in a small sample, and 2) patterns of correlation among loci and factors responsible for trait variation can create indirect associations between markers and traits where no causal relationship exists (known as population structure). The former can be managed by increasing sample size and the latter problem can be solved by validating identified QTL in an independent, but demographically similar population. If the same marker is highly associated across different traits and different populations, then it is very likely to be in strong linkage disequilibrium (LD) with the causal variant.

We performed GWAS using 50 K genotypes scored in 18,274 animals from 10 US beef cattle breeds with observations for twelve economically relevant traits (Tables 1). This large data set enabled us to perform an extensive comparative genome-wide association study in cattle (the largest GWAS in beef cattle ever performed), which had as its goal the identification of regions of the genome that account for at least 1% of genetic variation in these traits, and evaluation of genetic architecture of these traits among breeds. This analysis enabled discovery of several important across-breed and breed-specific large-effect pleiotropic or closely linked QTL.

**Table 1:** **Numbers of genotyped animals with deregressed estimated breeding values used as response variables for each trait in 10 US cattle breeds (ordered by sample size)**

Trait ^1^	Simmental	Angus	Hereford	Limousin	Red Angus	Gelbvieh	Brangus	Maine-Anjou	Shorthorn	Charolais
Birth weight	3930	3203	2735	2185	1711	1254	1316	573	324	200
Calving ease direct	3696	3180	1926	1687	1558	1249	1301	570	318	195
Calving ease maternal	3650	1965	1589	1669	1541	1242	1106	563	318	145
Carcass weight	3859	2448	-	1459	1704	1241	1126	565	238	143
Fat thickness	3347	3155	2311	-	1495	980	1060	171	230	128
Marbling	3338	3199	2242	1447	1510	955	1055	133	231	127
Mature weight	-	1321	2675	-	-	-	-	-	-	-
Ribeye muscle area	3326	3231	2509	1449	1500	943	1101	128	236	138
Weaning weight direct	3885	3191	2737	2150	1710	1248	1319	571	324	198
Weaning weight maternal	3767	2067	1898	1480	1659	1213	583	561	313	155
Yearling weight	3890	2755	2735	1780	1710	1247	1279	571	292	178
Yield grade	3460	-	-	1446	1523	1058	-	301	-	-
Total Animals	4124	3570	2779	2239	1761	1374	1328	574	328	200

^1^Birth weight: live weight at birth. Calving ease direct: the ability of a calf to be born unassisted because of its size and length of gestation. Calving ease maternal: the genetic ability of a dam for unassisted calving of her newborn because of her own pelvic size, her ability to relax the pelvis and the ability of her uterus to limit feral growth to a manageable size. Carcass weight: weight recorded just before the carcass enters the chilling room during the processing of finished cattle. Fat thickness: the amount of fat opposite the ribeye muscle at the cut surface between the 12^th^ and 13^th^ ribs. Marbling: the amount and distribution of intramuscular fat on the cut surface of the ribeye muscle between the 12^th^ and 13^th^ ribs. Mature weight: live body weight at maturity. Ribeye muscle area: area measurement on the cut surface of the ribeye muscle between the 12^th^ and 13^th^ ribs. Weaning weight direct: the pre-weaning growth ability of a calf. Weaning weight maternal: the milking and mothering ability of a dam for pre-weaning growth of a calf. Yearling weight: live weight at yearling. Yield grade: the estimated amount of boneless, closely trimmed retail cuts from the high-value parts of the carcass-the round, loin, rib, and chunk.

Gene enrichment analysis provided valuable information as to the biological functions of gene subsets and dominant pathways imposed in the trait of interest [4]. In addition to broad-spectrum GWAS, we used gene ontology (GO) tools to allocate biological roles and common pathways involved in the function of candidate genes within the identified pleiotropic QTL.

## Results and discussion

### Detected QTL and sample size

**Figure 1:**
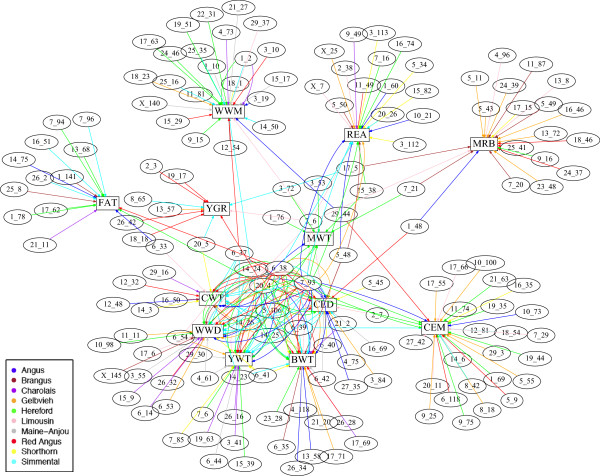
**The QTL network.** The genomic locations (BTA_Mb) and the trait(s) associated with each identified QTL. The traits are birth weight (BWT), calving ease direct (CED), calving ease maternal (CEM), carcass weight (CWT), marbling (MRB), mature weight (MWT), ribeye muscle area (REA), weaning weight direct (WWD), weaning weight maternal (WWM), yield grade (YGR) and yearling weight (YWT).

A total of 294 QTL (all considered to be of large-effect and defined as 1-Mb windows of the genome explaining ≥ 1% of additive genetic variance) were identified across twelve traits recorded in ten breeds (Table 2). Some of the identified QTL were associated with more than one trait (pleiotropic QTL) or segregating in more than one breed (across-breed QTL), which leave 159 QTL to be unique (Figure 1). In general, more QTL were identified in the populations (or traits) with bigger sample sizes (Table 2), which simply reflects the increasing statistical power in GWAS by increasing sample size [3, 5]. However, less QTL were identified in Angus and Limousin populations than expected by their sample sizes (Table 2), which could be due to different selection programs practiced in these breeds or due to the biased samples. Long-term directional selection changes the frequencies of causative variants toward fixation, which decreases the genetic variations and consequently the power of GWAS. This could be the case for these populations especially Angus that experienced long-term selection. Also, if the samples are not well distributed across the whole population and only a selected group of animals (usually those with high performances) are sampled then the power of GWAS would decrease due to lower genetic variation available in the selected samples (biased sample). This could be also a possibility with the samples collected in Angus and Limousin populations. Spencer et al. (2009) showed that the patterns of LD and the frequencies of causative variants (common versus rare alleles) also play important roles in the statistical power of GWAS [5].

**Table 2:** **Numbers of detected QTL (1-Mb windows of the genome explaining ≥ 1% of additive genetic variance) for each trait in 10 US cattle breeds (ordered by sample size)**

Trait ^1^	Simmental N = 4124	Angus N = 3570	Hereford N = 2779	Limousin N = 2239	Red Angus N = 1761	Gelbvieh N = 1374	Brangus N = 1328	Maine-Anjou N = 574	Shorthorn N = 328	Charolais N = 200	All breeds
Birth weight	7	4	5	2	5	5	2	2	3	2	37
Calving ease direct	5	4	3	3	5	4	1	2	2	0	29
Calving ease maternal	3	2	9	4	3	6	0	2	1	0	30
Carcass weight	9	3	-	2	4	3	2	1	2	1	27
Fat thickness	3	2	5	-	1	0	2	0	0	1	14
Marbling	0	1	1	3	3	6	5	0	1	0	20
Mature weight	-	1	9	-	-	-	-	-	-	-	10
Ribeye muscle area	3	4	4	3	1	2	2	0	4	1	24
Weaning weight direct	7	2	5	3	4	4	3	3	1	2	34
Weaning weight maternal	5	2	6	2	3	1	1	5	1	2	28
Yearling weight	8	2	6	1	3	2	1	3	2	3	31
Yield grade	3	-	-	1	5	1	-	0	-	-	10
All traits	53	27	53	24	37	34	19	18	17	12	294^2`^

^1^Birth weight: live weight at birth. Calving ease direct: the ability of a calf to be born unassisted because of its size and length of gestation. Calving ease maternal: the genetic ability of a dam for unassisted calving of her newborn because of her own pelvic size, her ability to relax the pelvis and the ability of her uterus to limit fetal growth to a manageable size. Carcass weight: weight recorded just before the carcass enters the chilling room during the processing of finished cattle. Fat thickness: the amount of fat opposite the ribeye muscle at the cut surface between the 12^th^ and 13^th^ ribs. Marbling: the amount and distribution of intramuscular fat on the cut surface of the ribeye muscle between the 12^th^ and 13^th^ ribs. Mature weight: live body weight at maturity. Ribeye muscle area: area measurement on the cut surface of the ribeye muscle between the 12^th^ and 13^th^ ribs. Weaning weight direct: the pre-weaning growth ability of a calf. Weaning weight maternal: the milking and mothering ability of a dam for pre-weaning growth of a calf. Yearling weight: live weight at yearling. Yield grade: the estimated amount of boneless, closely trimmed retail cuts from the high-value parts of the carcass-the round, loin, rib, and chunk.

^2^Some of the identified QTL were associated with more than one trait (pleiotropic QTL) or segregating in more than one breed (across-breed QTL), leaving a total of 159 unique QTL.

Details concerning support and magnitude of effect for each QTL associated with birth weight, calving ease direct, calving ease maternal, carcass weight, marbling, mature weight, ribeye muscle area, weaning weight direct, weaning weight maternal, yield grade and yearling weight in all ten cattle breeds are in Additional files 1, 2, 3, 4, 5, 6, 7, 8, 9, 10, 11 and 12, respectively. The identity of the most strongly associated SNP (denoted throughout as ‘lead-SNP’ and defined as the SNP with the highest posterior probability of inclusion (PPI) within the Bayesian GWAS model within the 1-Mb window most strongly associated with the QTL), its genomic position, its B allele (from the Illumina A/B calling system) frequency and the impact of that allele on the trait (increasing or decreasing) are also reported for each QTL in Additional files 1, 2, 3, 4, 5, 6, 7, 8, 9, 10, 11 and 12. In this study, a similar 1-Mb QTL associated with more than one trait considered as a pleiotropic QTL. However, it could be actually two different closely linked QTL. Further analyses using multi-variate models are needed to dissect pleiotropic QTL from closely linked QTL. However, when the B allele at lead-SNP is consistent for direction of affect across different breeds, which was the case for most identified lead-SNPs within identified QTL, then it is more likely to be pleiotropy because with linkage the B allele could detect the QTL1 + \QTL2+ haplotype (favorable alleles at two linked QTL) in one breed and the A allele could detect it in another. Additionally, most of the identified lead-SNPs are located in master regulator genes, which is further evidence for pleiotropy.

### Four pleiotropic QTL segregate in several phylogenetically distinct US cattle breeds

Among all identified QTL, four pleiotropic QTL were detected in two or more breeds (across-breed pleiotropic QTL). These pleiotropic QTL were identified on BTA6 at 37–42 Mb (varies across breeds but primarily detected at 38 Mb), on BTA7 at 93 Mb, on BTA14 at 23 – 26 Mb (primarily at 24 or 25 Mb) and on BTA20 at 4 Mb (Table 3). In general, the QTL on BTA6 had a larger impact than any of the other QTL and was associated with a greater number of traits in the greatest number of breeds (Table 3). This QTL is associated with body weights (birth, carcass, direct weaning, mature, and yearling weights), calving ease direct and weaning weight maternal, in all breeds except Angus. It explained more than 10% of additive genetic variance of birth weight in Hereford, Limousin, Red Angus and Simmental; of calving ease direct in Hereford; of carcass weight in Red Angus and Simmental; of mature weight in Hereford; of weaning weight direct in Limousin and Red Angus; and of yearling weight in Hereford, Limousin and Red Angus. The largest effect found for this QTL was for calving ease in Hereford where it explained 32% of additive genetic variance. The 1-Mb QTL region on BTA6 most strongly associated with the traits varied from 38 to 39 Mb and was not unique across breeds or traits. Further, there were six different lead-SNPs at 38 Mb and four different lead-SNPs at 39 Mb detected for different traits and breeds. However, the more frequently model-selected SNPs in this region were *rs81131471* at 38.91 Mb, *rs110834363* at 38.94 Mb and *rs81151923* at 39.26 Mb (near the boundary separating the 38 and 39 Mb windows). Many studies have pointed to the presence of QTL on BTA6 for body weights, growth and carcass traits [6–11], calving ease direct [12], milk traits [13–15], reproductive traits [16–18] and feed efficiency traits [8, 9, 19] in cattle. Three interesting genes located in this region have been suggested as positional candidate genes: 1) *LAP3* (leucine aminopeptidase 3), located at 38.57 - 38.60 Mb, which is an aminopeptidase which catalyzes the removal of N-terminal amino acids and is involved in protein maturation and degradation [15]; 2) *NCAPG* (non-SMC condensing I complex, subunit G), located at 38.74 - 38.81 Mb, which has a catalytic function in the mammalian condensin I complex and is important during mitotic cell division [20]; 3) *LCORL* (ligand dependent nuclear receptor corepressor-like), located at 38.84 - 38.99 Mb, which is associated with stature in human and cattle [21]. A nonsynonymous substitution in *NCAPG*, *NCAPG c.1326 T > G* that leads to the amino acid change p.Ile442Met in the NCAPG protein has been suggested as the candidate causative variant associated with prenatal [22] and postnatal growth traits [10, 20] in cattle. However, Bongiorni et al. (2012) has suggested that *LAP3* is the more probable gene to affect calving ease direct in Piedmontese cattle [12]. Whether the p.Ile442Met variant is responsible for the pleiotropic QTL on BTA6 detected in this study or if different mutation(s) in *NCAPG* or other genes are involved is currently unknown.

**Table 3:** **QTL (1-Mb windows of the genome explaining ≥ 1% of additive genetic variance) associated with more than one trait or segregating in more than one breed** ^**1**^

BTA_Mb ^2^	Start SNP	End SNP	No. SNP	Associated trait(s) ^3^	Segregating breed(s) ^4^
1_2	*rs110875985*	*rs43212498*	24	WWM	CHA, HER, SIM
1_48	*rs110988233*	*rs110340489*	24	CED, MRB	ANG, RAN
2_6	*rs29010906*	*rs41626743*	11	BWT, CED, MRB, REA, WWD, WWM, YGR	LIM
4_61	*rs109672663*	*rs43399326*	29	WWD, YWT	RDP
5_48	*rs29016809*	*rs41599228*	14	BWT, CED, REA	ANG, BRG
5_106	*rs109969273*	*rs110912524*	20	BWT, MWT, WWD, YWT	HER
6_33	*rs81127754*	*rs109867538*	23	CWT, FAT	ANG, LIM
6_37	*rs81128429*	*rs41577868*	27	CED, CWT, WWD, WWM, YWT	RAN, SIM
6_38	*rs29010895*	*rs110834363*	24	BWT, CED, CEM, CWT, FAT, MWT, REA, WWD, YWT	GVH, HER, LIM, RAN, SIM
6_39	*rs81139192*	*rs81129153*	27	BWT, CED, CEM, CWT, WWD, YWT	BSH, RAN, SIM
6_40	*rs81131541*	*rs29017603*	32	BWT, CED	BSH
6_41	*rs43463315*	*rs41651246*	31	BWT, WWD, YWT	BSH
6_42	*rs41651258*	*rs109415159*	27	YGR, BWT, CED	RDP
6_54	*rs109421050*	*rs43465018*	22	CWT, YWT	GVH, LIM
7_21	*rs43508635*	*rs43501063*	25	MRB, MWT	BRG, HER
7_93	*rs109819349*	*rs29009626*	11	BWT, CED, CEM, CWT, MWT, REA, WWD, YWT	ANG, BRG, GVH, HER, RAN, SIM
14_23	*rs41724672*	*rs81176130*	20	BWT, CWT, YWT	SIM
14_24	*rs110845339*	*rs41627956*	17	BWT, CED, CWT, WWD, WWM, YWT	GVH, SIM
14_25	*rs41627954*	*rs42298470*	21	BWT, CED, CWT, YWT	GVH, SIM
14_26	*rs81143942*	*rs81157855*	25	CED, CWT, WWD, YWT	BRG, SIM
15_38	*rs109164374*	*rs109550701*	18	MWT, REA	HER
17_5	*rs42927497*	*rs109541510*	27	MRB, YGR	BRG, SIM
20_4	*rs109377243*	*rs43094958*	28	BWT, CED, CEM, CWT, FAT, MWT, WWD, YGR, YWT	ANG, HER, RAN, SIM
20_5	*rs110348071*	*rs29020081*	29	CWT, YGR	BSH, SIM
21_2	*rs109456438*	*rs41644559*	16	BWT, CED	CHA, SIM
29_30	*rs110651226*	*rs109575701*	24	CWT, WWD, YWT	RDP
29_44	*rs110552089*	*rs109977592*	33	CEM, WWM	ANG, RAN

^1^A total of 294 QTL were identified which 27 of them were associated with more than trait or segregating in more than one breed leaving 159 unique QTL.

^2^Bovine chromosome and n^th^ 1 Mb window on the same chromosome starting at zero and based on the UMD3.1 assembly.

^3^The traits are shown as BWT (birth weight), CED (calving ease direct), CEM (calving ease maternal), CWT (carcass weight), MRB (marbling), MWT (mature weight), REA (ribeye muscle area), WWD (weaning weight direct), WWM (weaning weight maternal), YGR (yield grade) and YWT (yearling weight).

^4^The breeds are shown as AAN (Angus), BRG (Brangus), BSH (Shorthorn), CHA (Charolais), GVH (Gelbvieh), HER (Hereford), LIM (Limousin), RAN (Red Angus), RDP (Maine-Anjou) and SIM (Simmental).

The QTL on BTA7 at 93 Mb is pleiotropic and associated with birth weight, calving ease direct and maternal in Angus and Hereford; carcass weight in Angus and Simmental; mature weight in Hereford; ribeye muscle area in Angus, Gelbvieh, Hereford, Red Angus and Simmental; weaning weight direct and yearling weight in Angus, Hereford and Simmental. This is the most important pleiotropic QTL in Angus and is the largest-effect QTL associated with ribeye muscle area in several breeds. This QTL explained more than 16% of additive genetic variance in ribeye muscle area in Gelbvieh (Additional file 8). Associations of this QTL with composition of fatty acids in beef has also been reported [23, 24]. Only one lead-SNP (*rs110059753*) was detected for this QTL in all associated traits in Angus, Brangus, Hereford and Simmental animals. That SNP, located at 93.22 Mb, was also lead-SNP for a weaning weight direct QTL in Red Angus (Additional file 9). The effect of the B allele at this lead-SNP was in the same direction (increasing or decreasing) for the associated traits across different breeds, indicating that linkage phase between this lead-SNP and the causal mutation is conserved across breeds (Additional files 1, 2, 3, 4, 7, 8, 10 and 12) suggesting that the SNP is very close to the causal mutation. There is a very good candidate gene located close to this lead-SNP. *ARRDC3* (arresting domain containing 3), located at 93.24 - 93.25 Mb, is a member of the arrestin superfamily that regulates obesity in mice and human males [25, 26]. Arrestins are signaling proteins that control metabolism usually through desensitization of the beta-adrenergic receptors which are present on the surface of almost every type of mammalian cell. These receptors are stimulated physiologically by the neurotransmitter, norepinephrine and the adrenal medullary hormone, epinephrine [27]. It has been shown that oral administration of some beta-adrenergic agonists increases muscle and decreases fat accretion in cattle, pigs, poultry, and sheep [27, 28]. Consequently, if the casual mutation(s) associated with body weights and carcass traits can be established as influencing *ARRDC3* expression or activity, it could be considered a natural beta-adrenergic agonist in cattle. The physiological roles of *ARRDC3* in cattle are unknown.

The QTL on BTA14 at 24 – 26 Mb is segregating in Brangus, Gelbvieh and Simmental. This QTL was associated with five body weights (birth, carcass, and yearling weights; weaning weights direct and maternal) and calving ease in Gelbvieh and Simmental and explained 1 – 2% of additive genetic variance in carcass, weaning and yearling weights in Brangus. No unique lead-SNP was detected for this pleiotropic QTL in either the 24 or 25 Mb windows. Three different lead-SNPs at 24 Mb and four different lead-SNPs at 25 Mb (extending from 24.44 to 25.70 Mb) were detected in Gelbvieh and Simmental. This QTL region harbors *PLAG1* (pleiomorphic adenoma gene 1) which has been shown to be associated with stature in a Holstein × Jersey F2 cross [29]; carcass weight in Japanese Black cattle [30]; and early life body weight, peripubertal weight and growth in New Zealand Holstein–Friesian cattle [31]. Utsunomiya et al. (2013) reported a QTL on BTA14 in the vicinity of *PLAG1* associated with variation in birth weight in Nellore cattle suggesting that *PLAG1* may also be responsible for variation in body weights in *Bos indicus* cattle [32]. Our results suggest that mutations in *PLAG1* may also be important in Brangus, Gelbvieh and Simmental cattle. Whether the same mutation is associated with body weights in Brangus, Gelbvieh and Simmental or if different mutation(s) in *PLAG1* or nearby genes cause this variation is unclear.

The pleiotropic QTL on BTA20 at 4 Mb was segregating in several breeds including Angus, Shorthorn, Hereford, Red Angus and Simmental. This QTL was associated with most body weights and with calving ease direct in these breeds. The telomeric adjacent 1-Mb window (at 5 Mb) also explained 1.9 and 1.1% of additive genetic variance of carcass weight in Shorthorn and yield grade in Simmental, respectively (Additional files 4 and 11). This QTL explained more than 5% of additive genetic variance for birth, mature and yearling weights; weaning weight direct; calving ease and more than 18% of additive genetic variance of mature weight in Hereford (Additional file 7). Several lead-SNPs spanning the region from 4.50 to 4.75 Mb were identified. The lead-SNP *rs43350564*, located at 4.62 Mb, was the most frequent model-selected SNP within this region across the different traits and breeds. The effect of the B allele at this SNP was in the same direction for the associated traits across the different breeds, indicating that this SNP is in the same linkage phase with the casual mutation across breeds. This lead-SNP is a downstream gene variant (a sequence variant located 3’ of a gene) of *ERGIC1* (endoplasmic reticulum-Golgi intermediate compartment protein 1). ERGIC1 is a cycling membrane protein contributing to the membrane traffic and selective transport of cargo between the endoplasmic reticulum, the intermediate compartment, and the Golgi apparatus, which plays important roles in the organization and function of the early secretory pathway [33]. It is unlikely that protein sorting is the sole function of the ERGIC family, but other functions are less clear [33]. The window defining the pleiotropic QTL on BTA20 contains another interesting candidate gene, *SH3PXD2B* (SH3 and PX domain 2B) located at 4.01 - 4.14 Mb. The SH3PXD2B protein is essential for normal postnatal growth and development [34]. In two independent mouse models, the absence of SH3PXD2B was found to profoundly impair normal development causing runted growth, craniofacial and skeletal abnormalities, hearing impairment, glaucoma and the virtual absence of white adipose tissue [34, 35]. In human, SH3PXD2B deficiency is responsible for the development of Frank-Ter Haar syndrome, an autosomal-recessive disorder characterized by skeletal, cardiovascular, and eye abnormalities [35]. It has been also shown that SH3PXD2B plays an important role in cellular attachment and cell spreading [36].

None of these four pleiotropic QTL was associated with marbling in any breed or with any trait in Charolais. Besides these four pleiotropic QTL, several QTL were identified that were associated with more than one trait, but segregating in different breeds (Table 3).

### Breed-specific pleiotropic QTL

Besides the four pleiotropic QTL that were found to segregate in more than one breed, several breed-specific pleiotropic QTL (associated with more than one trait but segregating in only one breed) were identified (Table 3). A pleiotropic QTL on BTA2 at 6 Mb associated with birth weight, calving ease direct, marbling, rib eye area, weaning weight direct, weaning weight maternal, and yield grade was identified in Limousin. This QTL explained 4.7, 1.2, 5.4, 11.8, 1.4, 5.3 and 13.8% of additive genetic variance in these traits, respectively (Additional files 1, 2, 6, 8, 9, 10 and 11). The telomeric adjacent 1-Mb window (at 7 Mb) also explained 3.8% of additive genetic variance in calving ease direct in Limousin (Additional file 2). Two lead-SNPs (*rs110233897* and *rs41638273*) in close proximity, separated by less than 0.02 Mb (at 6.68 and 6.70 Mb, respectively), were detected. This QTL window harbors myostatin (*MSTN*) a major gene responsible for double-muscled phenotype in cattle [37]. This phenotype occurs at high frequency in some breeds of cattle such as Belgian Blue and Piedmontese [38]. There are numerous mutations in this gene including non-synonymous, missense, premature stop codons and frame-shift variants that are associated with different levels of change in muscle morphology [38]. A specific *MSTN* mutation identified as F94L, has been shown to be associated with increased muscle growth and beef traits in Limousin [39].

A pleiotropic QTL on BTA5 at 48 – 50 Mb associated with birth weight, calving ease direct, marbling and ribeye muscle area was identified in Brangus. Cumulatively (over three contiguous 1-Mb windows), this QTL explained 6.9, 5.2, 8.4 and 2.8% of additive genetic variance in these traits, respectively (Additional files 1, 2, 6 and 8). This QTL also associated with ribeye muscle area in Angus (Table 3). However, there was no overlap between identities of lead-SNPs in Angus and Brangus. This QTL window harbors some very good candidate genes. The lead-SNP *rs29016809*, detected for birth weight in Brangus (Additional file 1), is located in an intron of *HMGA2* (high mobility group protein A2). The HMGA proteins are architectural transcription factors that regulate transcription of a variety of genes and direct cellular growth, proliferation and differentiation [40]. *HMGA2* variants are associated with human height [41], body size in dogs [42] and horses [43], and meat quality and carcass traits in pigs [44]. *HMGA2* knockout mice had 40% of the mean body weight of controls [45]. The other lead-SNP, *rs109566520*, which was detected for calving ease direct in Brangus (Additional file 2), is located in an intron of *MSRB3* (methionine-S-sulphoxide reductase 3). Lee et al. (2012) have shown that methylation of *MSRB3* is associated in humans with changes in gestational age at birth [46]. Mutations in *MSRB3* are also associated with hereditary deafness and primary tooth development during infancy in human [47, 48]. The lead-SNP for ribeye muscle area in Brangus (*rs41657459*, Additional file 8) is located in an intron of *LEMD3* (LEM domain containing 3), which is responsible for bone density disorders [49]. Loss of function mutations in *LEMD3* result in increased bone density, namely osteopoikilosis in human [50]. Although many QTL associated with body weights, meat and milk traits have been reported in this region [6, 18, 51], no associations between traits and variants in *HMGA2*, *MSRB3* and *LEMD3* have been reported in cattle.

A pleiotropic QTL on BTA5 at 106 Mb in Hereford explained 2.6, 3.9, 2.0 and 4.9% of additive genetic variance in birth weight, mature weight, weaning weight direct and yearling weight, respectively (Additional files 1, 7, 9 and 12). Lead-SNP *rs41654528* located on BTA5 at 106.23 Mb was associated with birth weight, weaning weight direct and yearling weight while *rs110421124* located 0.04 Mb telomeric of *rs41654528* at 106.27 Mb was associated with mature weight in Hereford. Lead-SNP *rs41654528* is located in an intron of the Fructose-2,6-bisphosphotase TIGAR (TP53-induced glycolysis and apoptosis regulator). *TIGAR*, which also known as the chromosome 5 open reading frame or C12orf5 in human, is a recently identified fructose-2,6-bisphosphatase that contributes to regulation of glucose metabolism [52]. Knockdown of TIGAR increased glycolysis with elevated fructose-2,6-biphosphate levels and reduced myocyte apoptosis whereas overexpression of TIGAR reduced glucose utilization and increased myocyte apoptosis in mice [53]. There is a missense variant (*rs207837488*) within this gene, which causes a V94A substitution. That variant could be one of the potential candidate quantitative trait nucleotides for the body weight associations in Hereford.

Another breed-specific pleiotropic QTL was identified in Hereford on BTA15 at 38–39 Mb and was associated with mature weight and ribeye muscle area explaining 1.2 and 2.5% of additive genetic variance in each of these traits, respectively. There was no overlap in the identity of lead-SNPs within this QTL window. A pleiotropic QTL on BTA15 was reported in the range 37.96 to 54.29 Mb associated with carcass weight, mature height and ribeye muscle area in Angus [51].

Two breed-specific pleiotropic QTL associated with carcass, weaning and yearling weights were identified in Maine-Anjou, one on BTA4 at 61 Mb and the other on BTA29 at 30 Mb. The BTA4 QTL explained less than 2% of additive genetic variance in these three traits, while the BTA29 QTL explained 3.3, 1.7 and 3.8% of additive genetic variance (Additional files 4, 10 and 12). Lead-SNP *rs43400956* located on BTA4 at 61.73 Mb was detected for weaning weight direct and yearling weight in Maine-Anjou. This lead-SNP is located just 0.02 Mb telomeric of *SEPT7* (61.61 - 61.71 Mb), a member of the septin gene family. The septins are conserved GTP-binding proteins that form filaments during cell divisions or cytokinesis [54, 55]. The septins are also important in embryonic and nervous system development in animals [54]. There are several reported QTL in this region that are associated with body weights in Angus cattle [51, 56], carcass traits in Japanese Black cattle [57] and social behaviors in Charolais and Holstein [58]. Therefore, *SEPT7* may be a good candidate gene for the QTL associated with body weights in Maine-Anjou.

Lead-SNP *rs41651735* located on BTA29 at 30.70 Mb was also detected for carcass weight, weaning weight direct and yearling weight in Maine-Anjou. This lead-SNP is located in *KIRREL3* (kin of IRRE like 3), which is essential for the formation of synapses for cell-cell communication [59]. Variants in *KIRREL3* are associated with intellectual disability in human [60]. Many QTL associated with carcass, production, reproduction and behavior traits have been reported in different cattle breeds in this region [6, 18, 51].

### Trait-specific QTL

Only one trait-specific QTL (associated with only one trait but segregating in more than one breed) was identified and it was on BTA1 at 2 Mb (Table 3). It was associated with weaning weight maternal in Charolais, Hereford and Simmental and explained 1.3, 3.0 and 1.0% of additive genetic variance in these breeds, respectively (Additional file 10). The lead-SNP *rs109378326* at 2.52 Mb was detected for weaning weight maternal in Charolais and Simmental while *rs109205247* at 2.46 Mb was found in Hereford. The adjacent telomeric 1-Mb window to this QTL window (at 3 Mb) explained 0.7% of additive genetic variance in weaning weight maternal in Angus and Gelbvieh (data not shown). The milk production ability of a cow in terms of total energy available to the calf (comprising milk volume and composition) is a key factor affecting weaning weight in beef cattle. Many QTL associated with milk production traits (fat, protein and yield) have been reported in this region [61, 62]. McClure et al. (2010) also reported a QTL on BTA1 at 1–1.8 Mb associated with weaning weight maternal in Angus cattle [51]. *MRAP2* (melanocortin 2 receptor accessory protein), which is located in this QTL window encodes a melanocortin receptor-interacting protein that regulates trafficking and function of all members of the melanocortin receptor family in the adrenal gland [63]. Associations among polymorphisms in different melanocortin receptor family members and milk production traits in cattle and sheep have now been established [64, 65]. Loss of function of *MRAP2* is associated with mammalian obesity [66].

### Trait-breed specific QTL

Several trait-breed specific QTL (associated with only one trait and segregating in only one breed) were identified (Figure 1). Most of these QTL explained less than 2% of additive genetic variance of their corresponding trait in their respective breed. However, some of them explained a larger percentage of genetic variance. For example, a trait-breed specific QTL on BTA6 associated with birth weight explained 8.1% of additive genetic variance in Brangus (Additional file 1). The lead-SNP *rs110561712* on BTA6 at 35.56 Mb is an intronic variant within *CCSER1*. CCSER1 deficiency creates a cell division defect in human [67]. Experimental knockdown of *CCSER1* expression caused cytokinesis defects, multipolar mitosis and multinuclearity [67]. A trait-breed specific QTL associated with calving ease maternal (explaining more than 8% of additive genetic variance) was identified in Angus on BTA10 at 73 Mb (Additional file 3). The lead-SNP, *rs43638895* located on BTA10 at 73.83 Mb, is an intronic variant within *PRKCH* protein kinase C. Laramée et al. (2002) [68] showed that parathyroid-related peptide and parathyroid hormone, which have important roles in maternal-fetal calcium homeostasis, operate through activation of protein kinase C.

Five trait-breed specific QTL associated with marbling were identified in Gelbvieh on BTA5 at 11 Mb and 43 Mb; on BTA13 at 72 Mb; on BTA16 at 46 Mb; and on BTA24 at 39 Mb (Additional file 6). These cumulatively explained about 30% of additive genetic variance in marbling in Gelbvieh and the largest-effect QTL on BTA24 explained 10.5% of additive genetic variance (Additional file 6). There are few reported QTL in these regions associated with marbling in cattle. McClure et al. (2010) reported a similar QTL on BTA5 at 11 Mb associated with marbling score in Angus cattle [51]. Casas et al. (2000) reported overlapping QTL associated with fat thickness, ribeye muscle area and tenderness score on BTA5 [69].

Two adjacent trait-breed specific QTL on BTA3 at 112 and 113 Mb cumulatively explained 7.1% of additive genetic variance in ribeye muscle area in Shorthorn (Additional file 8). Another QTL on BTA5 at 34 Mb explained 3.9% of additive genetic variance of ribeye muscle area in Shorthorn (Additional file 8). A trait-breed specific QTL located on BTA26 at 42 Mb explaining 3.9% of additive genetic variance of yield grade was identified in Red Angus (Additional file 11). No QTL associated with yield grade have previously been reported in this region. We should point out that the reported trait-breed specific QTL are not validated QTL as they were identified only in one population and associated only with one trait. Independent populations are needed to validate these QTL.

### Genetic architecture of traits

**Figure 2:**
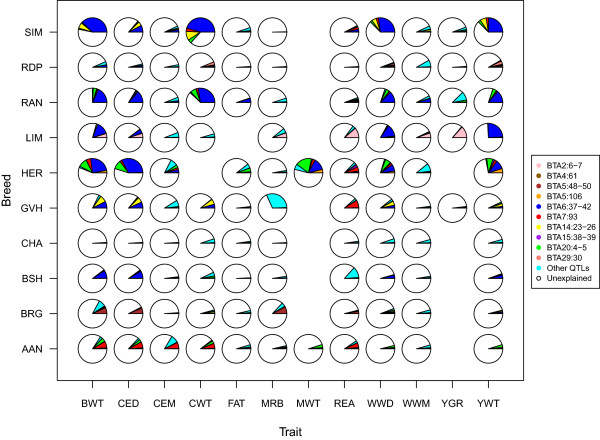
**Proportions of additive genetic variance explained by detected QTL.** The proportions of additive genetic variance explained by detected QTL (1-Mb windows of the genome explaining ≥ 1% of additive genetic variance) for each trait in 10 US cattle breeds. The traits are birth weight (BWT), calving ease direct (CED), calving ease maternal (CEM), carcass weight (CWT), marbling (MRB), mature weight (MWT), ribeye muscle area (REA), weaning weight direct (WWD), weaning weight maternal (WWM), yield grade (YGR) and yearling weight (YWT). The breeds are shown as AAN (Angus), BRG (Brangus), BSH (Shorthorn), CHA (Charolais), GVH (Gelbvieh), HER (Hereford), LIM (Limousin), RAN (Red Angus), RDP (Maine-Anjou) and SIM (Simmental).

Figure 2 shows the proportions of additive genetic variance explained by each of the across-breed and breed-specific pleiotropic QTL; and all other identified QTL. The four across-breeds pleiotropic QTL (on BTAs 6, 7, 14 and 20) and the breed-specific pleiotropic QTL explained most of the detected additive genetic variances of body weights (birth, carcass, yearling weights; and weaning weight direct) and calving ease direct in ten cattle breeds. The pleiotropic QTL explained a significant percentage of additive genetic variances of these traits cumulatively (more than a third of additive genetic variance of birth weight in Hereford and Simmental, calving ease direct in Herford, carcass weight in Red Angus and Simmental, mature weight in Hereford, weaning weight direct and yearling weight in Simmental, Figure 2). If the causal mutations of these pleiotropic QTL were found by further genotyping and statistical analyses, then knowledge of such mutations would create new opportunities for cattle breeders to select their animals with appropriate body weights for harvest or maternal purposes based on the few number of genes.

Different genetic architectures were observed for meat quality (fat thickness, marbling, ribeye muscle area and yield grade) and maternal (calving ease and weaning weight) traits. The detected QTL for these traits explained a lower percentage of genetic variances than those detected for body weights. This may reflect the fact that more selection pressure is on meat quality traits than growth traits in beef cattle, which may change the frequency of favorable allele(s) toward fixation, which decreases the genetic variance explained by a given QTL. Moderate body weights are more desirable for many producers because of increased calving problems and higher maintenance feed costs for animals with high body weights. The more likely reason could be the lack of power to detect QTL for meat quality traits due to less accurate data for carcass than growth traits. The accuracy of deregressed estimated breeding values (DEBV), used as response variables to estimate SNP effects, are generally lower for carcass traits versus growth traits due to lower number of observations and indirect measurement. For example, the average of reliability of DEBV for carcass weight, marbling and ribeye area in Angus was 0.41, 0.44 and 0.47, while it was 0.79, 0.69 and 0.70 for birth, weaning and yearling weights, respectively.

Most of the detected additive genetic variances for meat quality traits were explained by trait or trait-breed specific QTL and the pleiotropic QTL had less impact on these traits except for the pleotropic QTL on BTA 7 for ribeye muscle area and the Limousin-specific pleiotropic QTL on BTA 2. The QTL associated with ribeye muscle area had little overlap across the ten breeds. The pleiotropic QTL on BTA7 at 93 Mb was the only QTL associated with ribeye muscle area segregating in more than one breed (Angus, Gelbvieh, Hereford and Simmental). The pleiotropic QTL on BTA2 at 6 Mb explained 11.8% of additive genetic variance in ribeye muscle area in Limousin (Additional file 8) while a second QTL on BTA2 at 38 Mb explained 1.8% of additive genetic variance. Only ten large-effect QTL were identified for yield grade in the five breeds recorded for the trait. The QTL on BTA2 at 6 Mb in Limousin (pleiotropic QTL associated with many traits in Limousin) was the largest-effect QTL explaining 13.8% of additive genetic variance (Additional file 11). Five of these QTL were identified in Red Angus, three in Simmental and one in Gelbvieh and none overlapped (Additional file 11). There was also no overlap in marbling QTL across different breeds, which could be partly due to lack of power to detect all QTL associated with marbling in different breeds. Obviously not all QTL are discovered and those that are detected are different partly due to chance in different breeds. The QTL associated with weaning weight and calving ease maternal also had little overlap across the ten different breeds. The QTL on BTA1 at 2 Mb was the only QTL associated with weaning weight maternal segregating in more than one breed (Charolais, Hereford and Simmental, Additional file 10). The detected QTL explained less than 50% of additive genetic variances of the traits across all ten breeds (except for carcass weight in Simmental). This shows that a large number of loci of small effect are necessary to capture the remaining genetic variation as has been shown previously [70]. Here, we should acknowledge that the reported percentage of additive genetic variance explained for the identified QTL could be biased upward (or downward) due to influence of the prior used in the Bayesian regression model [71].

One might expect to find largely the same variants segregating in phylogenetically similar breeds due to their limited number of generations since divergence (see e.g., [72]). However, we did not find overwhelming evidence that this was the case since, for example, the first of the across-breed pleiotropic QTL was found to segregate in all breeds except Angus, yet Angus is closely related to other British breeds, namely Red Angus, Hereford and Shorthorn breeds. This result cannot be explained by small sample size since the Angus sample was the second largest studied. However, inadequate sample size may explain why QTL were not detected in Charolais, Maine-Anjou and Shorthorn. We suspect that resolution as to the concordance of causal mutations across breeds will not be resolved until multiple individuals from each of these breeds are sequenced to identify the extent of sharing of variation among breeds. Additional genotyping for variants discovered by sequencing and additional statistical analyses will be required to resolve identities of casual mutations. Elucidation of even a few of these mutations will create new opportunities for selection of animals with appropriate harvest body weights and meat quality specifications as well as for creation of maternal lines which could decrease dystocia and increase growth rate of calves in the US cattle population.

### Genes involved in ossification and in adipose tissue development are over-represented in the pleiotropic QTL

Four across-breed pleiotropic QTL (on BTAs 6, 7, 14 and 20), Limousin-specific pleiotropic QTL on BTA 2 and Hereford-specific pleiotropic QTL on BTA 5 were associated with the greater number of traits (these represent the core of the QTL network, Figure 1). Genes within these pleiotropic QTL along with the genes within Brangus-specific pleiotropic QTL (in total 83 genes, Additional file 13) were interrogated for GO (gene ontology) category and KEGG (Kyoto encyclopedia of genes and genomes) pathway enrichment using the web-based tool g:Profiler (http://biit.cs.ut.ee/gprofiler/) [73] GO analysis of the genes within these pleiotropic QTL showed significant enrichment for functional categories related to tissue development especially ossification and adipose tissue development (Table 4). Interestingly, all of the 8 pleiotropic QTL had genes involved in tissue development, where four of them include genes involved in the ossification process and 3 others involved our candidate genes (HMGA2, ARRDC3 and SH3PXD2B) in adipose tissue development (Table 4). The GO categories related to the cellular responses to the chemical stimulus, and the regulations of cation (especially calcium) transporter activities were also significantly over-represented in the pleiotropic QTL (Table 4). The pathway enrichment revealed that 5 genes (FGF6, FGF23, C-MOS, RRAS2, DUSP1) from 4 pleiotropic QTL (on BTAs 5, 14, 15 and 20) are significantly associated with the Mitogen-Activated Protein Kinase (MAPK) signaling pathway (KEGG:04010). The MAPK cascade is a highly conserved module that controls many cellular events from complex programs, such as embryogenesis, cell differentiation, cell proliferation, and cell death, to short-term changes required for homeostasis and acute hormonal responses [74].

**Table 4:** **List of significant gene ontology (GO) terms from GO analysis of genes that exist in the identified across-breed and breed-specific pleiotropic QTL**

GO ^1^	Term ID	p-value	Term name	Gene list	BTA_Mb ^2^
BP	GO:0009888	5.00E-02	Tissue development	SLC40A1, HMGA2, LEMD3, FGF6, FGF23, PKD2, SPP1, MEPE, IBSP, ARRDC3, IMPAD1, INSC, SH3PXD2B, NKX2-5	2_6, 5_48, 5_106, 6_38, 7_93, 14_25, 15_38, 20_4
BP	GO:0001503	3.33E-02	Ossification	FGF23, SPP1, MEPE, IBSP, IMPAD1, CT, CALC3	5_106, 6_38, 14_25, 15_38
BP	GO:0030279	3.25E-03	Negative regulation of ossification	FGF23, MEPE, CT, CALC3	5_106, 6_38, 15_38
BP	GO:0060612	2.50E-02	Adipose tissue development	HMGA2, ARRDC3, SH3PXD2B	5_48, 7_93, 20_4
BP	GO:0070887	2.54E-02	Cellular response to chemical stimulus	MSTN, HMGA2, LEMD3, FGF6, FGF23, PKD2, SPP1, IBSP, LYN, CT, CALC3, C5ORF41, STC2	2_6, 5_48, 5_106, 6_38, 15_38, 20_4, 29_30
BP	GO:2001259	2.50E-02	Positive regulation of cation channel activity	EFCAB4B, PKD2, NKX2-5	5_106, 6_38, 20_4
BP	GO:1901021	1.52E-02	Positive regulation of calcium ion transmembrane transporter activity	EFCAB4B, PKD2, NKX2-5	5_106, 6_38, 20_4
ke	KEGG:04010	5.00E-02	MAPK signaling pathway	FGF6, FGF23, C-MOS, RRAS2, DUSP1	5_106, 14_24, 15_38, 20_4

^1^BP: Biological process, ke: KEGG pathway.

^2^Bovine chromosome and n^th^ 1 Mb window on the same chromosome starting at zero and based on UMD3.1 assembly.

Some of the significant GO categories are not convincing to have pleiotropic roles (e. g. cellular responses to the chemical stimulus or the regulations of cation, Table 4) or some of the genes listed in the significant GO categories have a wider role than just that significant category (e. g. HMGA2 and ARRDC3 genes listed in adipose tissue GO term). Also, some of the significant GO categories do not include any candidate genes identified from association study in the pleiotropic QTL (e. g. ossification or MAPK signaling pathway, Table 4). This could be partly due to the GO analysis approach that we used in this study. Here, all genes within a 1-Mb were simply considered as significant genes. This assumption limits the analysis to find biological meaning.

## Conclusions

Although many quantitative trait loci (QTL) associated with economically important traits in beef cattle have been identified, not all of the genetic variation in these traits has been captured because of inadequate sample size and insufficient density of markers historically used in QTL mapping studies. Here, we took advantage of a high-density SNP assay that spans the genome at moderate resolution in conjunction with large sample size (18,274 animals from 10 US beef cattle breeds) for the identification of novel QTL and improvement of the resolution of the location of previously mapped QTL. We found a total of 159 unique large-effect QTL (defined as 1-Mb genome windows explaining more than 1% of additive genetic variance), where four QTL have pleiotropic effects (associated with more than one trait) and segregate in more than one breed. We also found pleiotropic QTL that segregate in single breeds. GO analysis revealed that genes involved in ossification and in adipose tissue development were over-represented in the identified pleiotropic QTL. Also, GO analysis showed that our identified pleiotropic QTL harbor genes that involved in the MAPK signaling pathway. Our results will improve understanding of the biology of growth and body composition in cattle.

## Methods

Animal Care and Use Committee approval was not required or obtained for data that were extracted from existing breed association databases. Blood, semen or hair samples collected on Angus, Hereford, Gelbvieh, Limousin, Simmental and Shorthorn animals for genotyping at the University of Missouri were collected under protocol 7505 approved by the University of Missouri Animal Care and Use Committee.

### Genotype and phenotype data

A total of 18,274 animals from 10 US cattle breeds (3,570 Black Angus, 1,328 Brangus, 200 Charolais, 1,374 Gelbvieh, 2,779 Hereford, 2,239 Limousin, 1,761 Red Angus, 328 Shorthorn, 574 Maine-Anjou and 4,124 Simmental) were genotyped with either BovineSNP50 BeadChip versions B or C (Illumina, San Diego, CA) or (less than 3%) with the BovineHD BeadChip (Illumina, San Diego, CA). For animals that were genotyped with the BovineHD BeadChip, genotypes for markers that were in common with the BovineSNP50 BeadChip were extracted. The marker quality control tests were performed using data available in the University of Missouri database at the time of analysis. Genotypes at a particular locus were filtered from further analysis according to the following criteria: average heterozygosity more than 0.52 in more than 10 breeds; average call rate less than 0.80 in more than 10 breeds; monomorphic in more than 7 breeds; minor allele frequency less than 0.001 and Hardy-Weinberg equilibrium test p-value less than 3 × 10^- 9^. Only markers that passed quality control and that were uniquely assigned to bovine autosomes or the X chromosome on the UMD3.1 assembly were used for analysis (54,555 markers). Missing genotypes (less than 3%) were replaced with the average value (on a 0–2 scale) for each SNP within each breed.

In total, twelve traits were analyzed (birth, carcass, mature and yearling weights; weaning weight and calving ease direct and maternal; fat thickness; marbling; ribeye muscle area and yield grade), however, some traits were recorded in only some breeds (Table 1). Expected progeny differences (EPD, which is one half of the estimated breeding value, EBV) and their Beef Improvement Federation (BIF) accuracies were obtained from each breed association for all genotyped animals and their sires and dams. The EPD were transformed to EBV by multiplying by 2 and corresponding reliabilities (R^2^) were obtained as *R*^2^ = 1 - (1 - *BIF* _ *Accuracy*)^2^.

Next, deregressed estimated breeding values (DEBV) were derived [75] and used as response variables to estimate SNP effects in a weighted analysis which used weights corresponding to the amount of information that was available for the estimation of each DEBV. This method results in DEBV that are free of parent average effects and the weights can be used to appropriately account for heterogeneous variance due to differences in reliabilities of individual and parent average EBV and therefore of corresponding DEBV. The proportion of additive genetic variance not explained by markers (parameter c of [75]) was assumed to be 0.40 [76–78] and heritabilities that were used to derive the weighting factors are in Table 5. The number of genotyped animals with DEBV varied among traits because some animals had no individual or offspring information contributing to their EPD or because some traits were recently introduced after some of the older animals had already had their progeny recorded. The numbers of genotyped animals with DEBV for each trait in each breed are in Table 1.

**Table 5:** **Trait heritabilities used to derive factors for weighted analyses of deregressed estimated breeding values in 10 US cattle breeds**

Trait ^1^	Angus	Brangus	Charolais	Gelbvieh	Hereford	Limousin	Maine-Anjou	Red Angus	Shorthorn	Simmental
Birth weight	0.42	0.42	0.49	0.42	0.43	0.42	0.42	0.42	0.36	0.42
Calving ease direct	0.18	0.23	0.24	0.12	0.10	0.12	0.12	0.12	0.14	0.12
Calving ease maternal	0.12	0.12	0.16	0.13	0.10	0.13	0.13	0.13	0.14	0.13
Carcass weight	0.40	0.27	0.21	0.40	-	0.40	0.40	0.40	0.32	0.40
Fat thickness	0.34	0.35	0.26	0.35	0.30	-	0.35	0.35	0.35	0.35
Marbling	0.45	0.23	0.36	0.54	0.26	0.54	0.54	0.54	0.28	0.54
Mature weight	0.55	-	-	-	0.10	-	-	-	-	-
Ribeye muscle area	0.51	0.39	0.34	0.46	0.26	0.46	0.46	0.46	0.33	0.46
Weaning weight direct	0.20	0.24	0.25	0.30	0.20	0.30	0.30	0.30	0.23	0.30
Weaning weight maternal	0.14	0.12	0.14	0.15	0.10	0.14	0.15	0.15	0.06	0.15
Yearling weight	0.45	0.49	0.31	0.29	0.36	0.29	0.29	0.29	0.36	0.29
Yield grade	-	-	-	0.40	-	0.40	0.40	0.40	-	0.40

^1^Birth weight: live weight at birth. Calving ease direct: the ability of a calf to be born unassisted because of its size and length of gestation. Calving ease maternal: the genetic ability of a dam for unassisted calving of her newborn because of her own pelvic size, her ability to relax the pelvis and the ability of her uterus to limit fetal growth to a manageable size. Carcass weight: weight recorded just before the carcass enters the chilling room during the processing of finished cattle. Fat thickness: the amount of fat opposite the ribeye muscle at the cut surface between the 12^th^ and 13^th^ ribs. Marbling: the amount and distribution of intramuscular fat on the cut surface of the ribeye muscle between the 12^th^ and 13^th^ ribs. Mature weight: live body weight at maturity. Ribeye muscle area: area measurement on the cut surface of the ribeye muscle between the 12^th^ and 13^th^ ribs. Weaning weight direct: the pre-weaning growth ability of a calf. Weaning weight maternal: the milking and mothering ability of a dam for pre-weaning growth of a calf. Yearling weight: live weight at yearling. Yield grade: the estimated amount of boneless, closely trimmed retail cuts from the high-value parts of the carcass-the round, loin, rib, and chunk.

### Statistical model

In this study, all 54,555 SNP markers were simultaneously considered as predictors of the response variables in order to estimate partial SNP effects. The “Bayes-B” method [79] which fits a mixture model in which non-zero SNP effects are drawn from distributions with marker specific variances and some known fraction of markers (π) have no effect on the trait was used to estimate marker effects. For each trait, the following model was fit to estimate marker effects:


where **y** is the vector of observations (i.e., DEBV); **b** is the vector of fixed effects which comprised only the population mean because DEBV are free of systematic environmental effects such as herds, years and seasons of data origin; **u** is a vector of random marker substitution effects, where element j of **u** has effect greater than zero (with probability 1 - π) or effect equal to zero (with probability π) as described by [80]; **X** and **Z** are design matrices which relate observations to the fixed and marker effects, respectively, with each element of **Z** representing an allelic state (i.e., centered number of B alleles from the Illumina A/B calling system); and **e** is the vector of random residuals ~ N(0, ) where **D** is a diagonal matrix whose inverse elements are the weights described by [75]. The DEBV for maternal traits (calving ease and weaning weight maternal) were derived from EBV reported by respective breed association similar to other traits. In this study, parameter π was set to 0.99 for all analyses. MCMC methods with 41,040 iterations were used to obtain samples of marker effects and variances after discarding the first 1,000 samples to allow for burn-in. The estimates of genetic and residual variances for constructing priors of genetic and residual scale parameters for Bayes-B analysis [80], were obtained from preliminary Bayes-C analyses with π = 0.95 [81], which is less sensitive to prior assumptions than Bayes-B.

For each 40^th^ iteration of the post burn-in chain (1,000 samples in total), sampled values for the effects of the SNPs within each 1-Mb window were used to compute samples of the direct genomic breeding value (DGV) of every animal for that window (by multiplying the number of copies of B alleles by the sample of their corresponding SNP effect, and summing these values over all marker loci located within the 1-Mb window). The variance of window DGV across all animals within the breed was then used to obtain a sample of the additive genetic variance for that window. The percentage of additive genetic variance explained by each 1-Mb window was calculated as the proportion of the 1-Mb window variance for that sample relative to the sample in the same iteration of the whole genome additive genetic variance. Any 1-Mb window for which the posterior mean percentage of additive genetic variance explained was ≥ 1% (~25 fold greater than the expected value of 0.04% for each of 2,677 1-Mb windows genome-wide assuming a polygenic model for which all genomic regions explain the same amount of variance) was selected as a window containing (or defining) a large-effect QTL. Those QTL that were associated with at least two traits in more than one breed were considered to be pleiotropic. The posterior probability of inclusion (PPI) for a given window, which is the proportion of samples in which at least one SNP from a given window was included in the model with a non-zero effect, was used for significance testing [82]. All analyses were performed using GenSel software [82].

Individual 1-Mb windows that explained the largest proportions of additive genetic variation were visualized in GBrowse [83] for detailed inspection of the chromosomal region containing the 1-Mb window. The overlapping QTL for a given 1-Mb window were obtained from cattle QTLdb (http://www.animalgenome.org/cgi-bin/gbrowse/bovine/). The SNP with the highest PPI within a given 1-Mb QTL window was selected as the lead-SNP or most strongly associated SNP for that QTL window. Further information for strongly associated SNP was obtained using NCBI dbSNP (http://www.ncbi.nlm.nih.gov/snp/) and Ensemble (http://www.ensembl.org/) databases. Gene searches were performed for these genomic regions using the NCBI gene database (http://www.ncbi.nlm.nih.gov/gene/). The sfdp algorithm from Graphviz software was used to draw the QTL network [84]. GO term enrichment analysis was performed for gene sets that existed within the pleiotropic QTL (those with available GO ID, Additional file 13) over all known genes in the GO database using the web tool g:Profiler (http://biit.cs.ut.ee/gprofiler/) [73] with Bonferroni corrected *p*-value cut off 0.05. Only GO terms from the categories biological processes and KEGG pathway enrichments were retained.

### Availability of supporting data

All association results have been deposited in the AnimalQTLdb (http://www.animalgenome.org/cgi-bin/QTLdb/BT/qabstract?PUBMED_ID=ISU0069).

## Electronic supplementary material

Additional file 1: **Large-effect QTL associated with birth weight in 10 cattle breeds.** (DOCX 44 KB)

Additional file 2: **Large-effect QTL associated with calving ease direct in 10 cattle breeds.** (DOCX 40 KB)

Additional file 3: **Large-effect QTL associated with calving ease maternal in 10 cattle breeds.** (DOCX 41 KB)

Additional file 4: **Large-effect QTL associated with carcass weight in 9 cattle breeds.** (DOCX 40 KB)

Additional file 5: **Large-effect QTL associated with fat thickness in 9 cattle breeds.** (DOCX 35 KB)

Additional file 6: **Large-effect QTL associated with marbling in 10 cattle breeds.** (DOCX 37 KB)

Additional file 7: **Large-effect QTL associated with mature weight in 2 cattle breeds.** (DOCX 33 KB)

Additional file 8: **Large-effect QTL associated with ribeye muscle area in 10 cattle breeds.** (DOCX 39 KB)

Additional file 9: **Large-effect QTL associated with weaning weight direct in 10 cattle breeds.** (DOCX 43 KB)

Additional file 10: **Large-effect QTL associated with weaning weight maternal in 10 cattle breeds.** (DOCX 41 KB)

Additional file 11: **Large-effect QTL associated with yield grade in 5 cattle breeds.** (DOCX 33 KB)

Additional file 12: **Large-effect QTL associated with yearling weight in 10 cattle breeds.** (DOCX 42 KB)

Additional file 13: **List of the genes within the pleiotropic QTL used for gene ontology analysis.** (XLSX 51 KB)

## Abbreviations

BIF: Beef Improvement Federation
DEBV: Deregressed estimated breeding values
DGV: Direct genomic breeding value
EPD: Expected progeny differences
GO: Gene ontology
GWAS: Genome-wide association studies
KEGG: Kyoto encyclopedia of genes and genomes
LD: Linkage disequilibrium
Mb: Megabases
MCMC: Markov chain Monte Carlo
PPI: Posterior probability of inclusion
QTL: Quantitative trait loci.
