# Supplementary material for: Large-effect pleiotropic or closely linked QTL segregate within and across ten US cattle breeds
Source: BMC Genomics. 2014 Jun 6;15(1):442. doi: 10.1186/1471-2164-15-442 (PMC4102727; doi:10.1186/1471-2164-15-442)
Supplement: Supplementary file 1 — Additional file 1: Large-effect QTL associated with birth weight in 10 cattle breeds. (DOCX 44 KB) [file 12864_2014_6256_MOESM1_ESM.docx]

**Table S1. Large-effect QTL associated with birth weight in 10 cattle breeds.**

| BTA_Mb^1^ | Start SNP | End SNP | No. SNP | Breed | %V_A_ | PPI^2^ | Lead SNP^3^ | Position (bp) | SNP Effect^4^ | Frequency^4^ |
| --- | --- | --- | --- | --- | --- | --- | --- | --- | --- | --- |
| 2_6 | *rs29010906* | *rs41626743* | 11 | Limousin | 4.67 | 0.99 | *rs41638273* | 6,700,805 | + | 0.79 |
| 4_118 | *rs110450060* | *rs42540403* | 27 | Maine-Anjou | 3.58 | 0.85 | *rs109276379* | 118,127,054 | + | >0.99 |
| 5_48 | *rs29016809* | *rs41599228* | 14 | Brangus | 6.32 | 0.86 | *rs29016809* | 48,080,259 | - | 0.31 |
| 5_106 | *rs109969273* | *rs110912524* | 20 | Hereford | 2.67 | 1.00 | *rs41654528* | 106,230,591 | - | 0.36 |
| 6_35 | *rs41595128* | *rs108983635* | 14 | Brangus | 8.08 | 0.96 | *rs110561712* | 35,555,247 | - | 0.86 |
| 6_38 | *rs29010895* | *rs110834363* | 24 | Gelbvieh | 6.96 | 1.00 | *rs81147999* | 38,576,012 | - | 0.28 |
|  |  |  |  | Hereford | 24.11 | 1.00 | *rs81131471* | 38,914,175 | + | 0.93 |
|  |  |  |  | Limousin | 12.98 | 1.00 | *rs81131471* | 38,914,175 | + | 0.78 |
|  |  |  |  | Red Angus | 15.35 | 1.00 | *rs110834363* | 38,939,012 | + | 0.47 |
|  |  |  |  | Simmental | 21.66 | 1.00 | *rs81131471* | 38,914,175 | + | 0.94 |
| 6_39 | *rs81139192* | *rs81129153* | 27 | Red Angus | 2.43 | 0.75 | *rs81151923* | 39,257,620 | + | 0.56 |
|  |  |  |  | Shorthorn | 4.93 | 0.85 | *rs81151923* | 39,257,620 | + | 0.65 |
|  |  |  |  | Simmental | 15.76 | 1.00 | *rs29026121* | 39,216,868 | + | 0.21 |
| 6_40 | *rs81131541* | *rs29017603* | 32 | Shorthorn | 1.53 | 0.55 | *rs81167259* | 40,922,391 | - | 0.64 |
| 6_41 | *rs43463315* | *rs41651246* | 31 | Shorthorn | 1.88 | 0.59 | *rs43459713* | 41,795,944 | + | 0.70 |
| 6_42 | *rs41651258* | *rs109415159* | 27 | Maine-Anjou | 1.62 | 0.63 | *rs43462195* | 42,609,559 | + | 0.90 |
| 7_93 | *rs109819349* | *rs29009626* | 11 | Angus | 7.01 | 1.00 | *rs110059753* | 93,218,452 | - | 0.30 |
|  |  |  |  | Hereford | 5.01 | 1.00 | *rs110059753* | 93,218,452 | - | 0.46 |
|  |  |  |  | Red Angus | 1.40 | 0.92 | *rs41625563* | 93,073,890 | - | 0.24 |
|  |  |  |  | Simmental | 1.18 | 0.97 | *rs110059753* | 93,218,452 | - | 0.64 |
| 13_58 | *rs41630667* | *rs43166604* | 22 | Angus | 1.32 | 0.99 | *rs110174530* | 58,285,197 | + | 0.50 |
| 14_23 | *rs41724672* | *rs81176130* | 20 | Simmental | 1.39 | 0.93 | *rs41628383* | 23,853,811 | - | 0.68 |
| 14_24 | *rs110845339* | *rs41627956* | 17 | Gelbvieh | 2.47 | 0.91 | *rs42649775* | 24,437,778 | - | 0.30 |
|  |  |  |  | Simmental | 1.26 | 0.98 | *rs41724332* | 24,643,266 | - | 0.23 |
| 14_25 | *rs41627954* | *rs42298470* | 21 | Gelbvieh | 4.92 | 0.99 | *rs29021334* | 25,612,510 | - | 0.38 |
|  |  |  |  | Simmental | 3.16 | 1.00 | *rs41627954* | 25,107,556 | - | 0.81 |
| 17_69 | *rs42275607* | *rs42288152* | 22 | Charolais | 1.65 | 0.21 | *rs109813560* | 69,030,893 | + | 0.65 |
| 17_71 | *rs109890790* | *rs41853809* | 29 | Gelbvieh | 1.15 | 0.85 | *rs110295933* | 71,231,159 | + | 0.58 |
| 20_4 | *rs109377243* | *rs43094958* | 28 | Angus | 3.61 | 1.00 | *rs43350564* | 4,618,689 | + | 0.45 |
|  |  |  |  | Hereford | 9.81 | 1.00 | *rs43349755* | 4,746,836 | + | 0.52 |
|  |  |  |  | Red Angus | 3.85 | 1.00 | *rs43350564* | 4,618,689 | + | 0.39 |
|  |  |  |  | Simmental | 1.12 | 0.95 | *rs43350564* | 4,618,689 | + | 0.21 |
| 21_2 | *rs109456438* | *rs41644559* | 16 | Charolais | 1.02 | 0.15 | *rs110887378* | 2,381,941 | - | 0.39 |
| 21_20 | *rs109222589* | *rs41640669* | 23 | Gelbvieh | 1.27 | 0.77 | *rs110218532* | 20,300,789 | + | 0.55 |
| 23_28 | *rs109756967* | *rs41622011* | 25 | Hereford | 1.63 | 0.98 | *rs109756967* | 28,021,047 | - | 0.69 |
| 26_28 | *rs41843046* | *rs41624190* | 23 | Red Angus | 1.02 | 0.82 | *rs29017020* | 28,544,401 | - | 0.66 |
| 26_34 | *rs108940561* | *rs110792748* | 22 | Angus | 1.27 | 0.99 | *rs41567908* | 34,800,551 | - | 0.38 |

^1^Bovine chromosome and n^th^ 1 Mb window on the same chromosome starting at zero and based on the UMD3.1 assembly.

^2^Posterior probability of inclusion (the proportion of MCMC samples in which SNP within the window had non-zero additive genetic variance).

^3^SNP with the highest posterior probability of inclusion within the window.

^4^The B alleles from the Illumina A/B calling system.
