# Supplementary material for: Large-effect pleiotropic or closely linked QTL segregate within and across ten US cattle breeds
Source: BMC Genomics. 2014 Jun 6;15(1):442. doi: 10.1186/1471-2164-15-442 (PMC4102727; doi:10.1186/1471-2164-15-442)
Supplement: Supplementary file 2 — Additional file 2: Large-effect QTL associated with calving ease direct in 10 cattle breeds. (DOCX 40 KB) [file 12864_2014_6256_MOESM2_ESM.docx]

**Table S2.** **Large-effect QTL associated with calving ease direct in 10 cattle breeds.**

| BTA_Mb^1^ | Start SNP | End SNP | No. SNP | Breed | %V_A_ | PPI^2^ | Lead SNP^3^ | Position (bp) | SNP Effect^4^ | Frequency^4^ |
| --- | --- | --- | --- | --- | --- | --- | --- | --- | --- | --- |
| 1_48 | *rs110988233* | *rs110340489* | 24 | Red Angus | 1.08 | 0.87 | *rs29024583* | 48,425,887 | + | 0.51 |
| 2_6 | *rs29010906* | *rs41626743* | 11 | Limousin | 1.24 | 0.37 | *rs41638273* | 6,700,805 | - | 0.79 |
| 2_7 | *rs109947846* | *rs42272790* | 21 | Limousin | 3.79 | 0.79 | *rs29018577* | 7,086,105 | - | 0.88 |
| 3_84 | *rs41571250* | *rs43710449* | 26 | Gelbvieh | 1.12 | 0.83 | *rs42908720* | 84,195,302 | + | 0.37 |
| 4_75 | *rs110091920* | *rs41654149* | 25 | Angus | 1.09 | 0.90 | *rs43406164* | 75,951,231 | - | 0.37 |
| 5_45 | *rs41614295* | *rs110004392* | 17 | Shorthorn | 1.10 | 0.50 | *rs81161030* | 45,420,592 | - | 0.64 |
| 5_48 | *rs29016809* | *rs41599228* | 14 | Brangus | 4.54 | 0.35 | *rs109566520* | 48,633,731 | + | 0.26 |
| 6_37 | *rs81128429* | *rs41577868* | 27 | Red Angus | 1.38 | 0.76 | *rs81129753* | 37,526,622 | + | 0.43 |
| 6_38 | *rs29010895* | *rs110834363* | 24 | Gelbvieh | 6.38 | 1.00 | *rs81147999* | 38,576,012 | + | 0.28 |
|  |  |  |  | Hereford | 31.69 | 1.00 | *rs81131471* | 38,914,175 | - | 0.93 |
|  |  |  |  | Limousin | 3.99 | 0.95 | *rs109447543* | 38,715,250 | - | 0.33 |
|  |  |  |  | Red Angus | 10.17 | 1.00 | *rs81128660* | 38,464,203 | - | 0.61 |
|  |  |  |  | Simmental | 1.67 | 0.75 | *rs110834363* | 38,939,012 | - | 0.55 |
| 6_39 | *rs81139192* | *rs81129153* | 27 | Red Angus | 1.73 | 0.66 | *rs81151923* | 39,257,620 | - | 0.57 |
|  |  |  |  | Simmental | 5.16 | 1.00 | *rs81165346* | 39,556,588 | - | 0.78 |
| 6_40 | *rs81131541* | *rs29017603* | 32 | Shorthorn | 6.32 | 0.83 | *rs81167259* | 40,922,391 | + | 0.63 |
| 6_42 | *rs41651258* | *rs109415159* | 27 | Maine-Anjou | 1.01 | 0.58 | *rs43462195* | 42,609,559 | - | 0.90 |
| 7_93 | *rs109819349* | *rs29009626* | 11 | Angus | 6.76 | 1.00 | *rs110059753* | 93,218,452 | + | 0.30 |
|  |  |  |  | Hereford | 2.36 | 0.93 | *rs110059753* | 93,218,452 | + | 0.45 |
| 14_24 | *rs110845339* | *rs41627956* | 17 | Gelbvieh | 1.85 | 0.89 | *rs42649775* | 24,437,778 | + | 0.30 |
| 14_25 | *rs41627954* | *rs42298470* | 21 | Gelbvieh | 3.84 | 1.00 | *rs41627953* | 25,307,116 | + | 0.29 |
|  |  |  |  | Simmental | 2.84 | 1.00 | *rs41627954* | 25,107,556 | + | 0.82 |
| 14_26 | *rs81143942* | *rs81157855* | 25 | Simmental | 1.07 | 0.96 | *rs41627964* | 26,450,034 | - | 0.23 |
| 16_69 | *rs109480845* | *rs42346321* | 25 | Maine-Anjou | 1.52 | 0.74 | *rs81111349* | 69,167,269 | - | 0.52 |
| 20_4 | *rs109377243* | *rs43094958* | 28 | Angus | 2.82 | 1.00 | *rs43350564* | 4,618,689 | - | 0.45 |
|  |  |  |  | Hereford | 9.90 | 1.00 | *rs43349755* | 4,746,836 | - | 0.53 |
|  |  |  |  | Red Angus | 1.29 | 0.96 | *rs29015626* | 4,567,765 | - | 0.74 |
| 21_2 | *rs109456438* | *rs41644559* | 16 | Simmental | 1.01 | 0.98 | *rs41644559* | 2,985,827 | + | 0.51 |
| 27_35 | *rs41572913* | *rs109612018* | 22 | Angus | 1.05 | 0.98 | *rs110127872* | 35,144,687 | - | 0.05 |

^1^Bovine chromosome and n^th^ 1 Mb window on the same chromosome started at zero and based on the UMD3.1 assembly.

^2^Posterior probability of inclusion (the proportion of MCMC samples in which SNP within the window had non-zero additive genetic variance).

^3^SNP with the highest posterior probability of inclusion within the window.

^4^The B alleles from the Illumina A/B calling system.
