# Supplementary material for: Large-effect pleiotropic or closely linked QTL segregate within and across ten US cattle breeds
Source: BMC Genomics. 2014 Jun 6;15(1):442. doi: 10.1186/1471-2164-15-442 (PMC4102727; doi:10.1186/1471-2164-15-442)
Supplement: Supplementary file 3 — Additional file 3: Large-effect QTL associated with calving ease maternal in 10 cattle breeds. (DOCX 41 KB) [file 12864_2014_6256_MOESM3_ESM.docx]

**Table S3.** **Large-effect QTL associated with calving ease maternal in 10 cattle breeds.**

| BTA_Mb^1^ | Start SNP | End SNP | No. SNP | Breed | %V_A_ | PPI^2^ | Lead SNP^3^ | Position (bp) | SNP Effect^4^ | Frequency^4^ |
| --- | --- | --- | --- | --- | --- | --- | --- | --- | --- | --- |
| 1_69 | *rs109544617* | *rs109811771* | 24 | Hereford | 1.01 | 0.58 | *rs29023069* | 69,138,050 | - | 0.59 |
| 5_9 | *rs43428684* | *rs42071421* | 24 | Red Angus | 1.32 | 0.88 | *rs41621912* | 9,806,599 | + | 0.23 |
| 5_55 | *rs41571380* | *rs110425294* | 16 | Gelbvieh | 1.06 | 0.70 | *rs41637710* | 55,378,288 | + | 0.61 |
| 6_38 | *rs29010895* | *rs110834363* | 24 | Hereford | 2.62 | 0.85 | *rs81131480* | 38,869,785 | - | 0.19 |
| 6_39 | *rs81139192* | *rs81129153* | 27 | Simmental | 1.65 | 0.95 | *rs81151923* | 39,257,620 | - | 0.59 |
| 6_118 | *rs43490816* | *rs43497840* | 26 | Red Angus | 1.18 | 0.89 | *rs109097693* | 118,579,602 | - | 0.79 |
| 7_29 | *rs41669611* | *rs109523073* | 21 | Limousin | 1.01 | 0.93 | *rs41627667* | 29,775,999 | - | 0.40 |
| 7_93 | *rs109819349* | *rs29009626* | 11 | Angus | 6.71 | 1.00 | *rs110059753* | 93,218,452 | + | 0.29 |
|  |  |  |  | Hereford | 2.23 | 0.89 | *rs110059753* | 93,218,452 | + | 0.45 |
| 8_18 | *rs109794673* | *rs110133763* | 26 | Gelbvieh | 1.14 | 0.76 | *rs109346659* | 18,759,713 | + | 0.63 |
| 8_42 | *rs109860247* | *rs41578462* | 25 | Simmental | 1.04 | 0.98 | *rs42698410* | 42,405,548 | - | 0.56 |
| 9_25 | *rs29013870* | *rs41609161* | 22 | Gelbvieh | 1.32 | 0.78 | *rs41656020* | 25,707,960 | - | 0.59 |
| 9_75 | *rs41578299* | *rs43705532* | 13 | Hereford | 1.55 | 0.73 | *rs43705532* | 75,932,080 | + | 0.66 |
| 10_73 | *rs42453444* | *rs41656323* | 20 | Angus | 8.64 | 0.53 | *rs43638895* | 73,828,302 | - | <0.01 |
| 10_100 | *rs81116755* | *rs29014597* | 26 | Gelbvieh | 1.41 | 0.89 | *rs110704616* | 100,943,686 | + | 0.55 |
| 11_74 | *rs109669210* | *rs81118348* | 17 | Hereford | 1.21 | 0.72 | *rs109949450* | 74,079,196 | + | 0.39 |
| 12_81 | *rs110576622* | *rs109434250* | 22 | Maine-Anjou | 1.06 | 0.59 | *rs109185699* | 81,642,369 | + | 0.24 |
| 14_6 | *rs81146812* | *rs81137124* | 33 | Maine-Anjou | 1.10 | 0.63 | *rs109634189* | 6,295,969 | + | 0.42 |
| 16_35 | *rs81111317* | *rs41579660* | 17 | Shorthorn | 1.09 | 0.56 | *rs41579673* | 35,317,388 | - | 0.76 |
| 17_55 | *rs29010166* | *rs110364620* | 27 | Gelbvieh | 1.09 | 0.79 | *rs109034521* | 55,816,005 | + | 0.60 |
| 17_66 | *rs41845288* | *rs109954806* | 28 | Limousin | 1.87 | 1.00 | *rs41852077* | 66,790,999 | - | 0.78 |
| 18_54 | *rs109907036* | *rs41893299* | 26 | Simmental | 1.15 | 0.99 | *rs110875944* | 54,376,451 | - | 0.54 |
| 19_35 | *rs109843005* | *rs43031950* | 28 | Hereford | 1.25 | 0.73 | *rs110886371* | 35,755,815 | + | 0.67 |
| 19_44 | *rs41577539* | *rs41917260* | 26 | Hereford | 1.71 | 0.85 | *rs110855079* | 44,216,479 | - | 0.43 |
| 20_4 | *rs109377243* | *rs43094958* | 28 | Hereford | 3.01 | 0.94 | *rs41932453* | 4,518,051 | + | 0.36 |
| 20_11 | *rs110815453* | *rs29025776* | 25 | Limousin | 1.14 | 0.95 | *rs43059073* | 11,803,942 | + | 0.52 |
| 21_63 | *rs41619857* | *rs110505514* | 26 | Hereford | 1.46 | 0.73 | *rs41626279* | 63,955,841 | + | 0.21 |
| 27_42 | *rs42135519* | *rs109395911* | 25 | Limousin | 1.33 | 0.99 | *rs110224190* | 42,344,117 | - | 0.58 |
| 29_3 | *rs42764618* | *rs41571007* | 17 | Gelbvieh | 1.18 | 0.76 | *rs42764618* | 3,074,352 | - | 0.69 |
| 29_44 | *rs110552089* | *rs109977592* | 33 | Red Angus | 1.26 | 0.85 | *rs41586223* | 44,196,154 | + | 0.88 |

^1^Bovine chromosome and n^th^ 1 Mb window on the same chromosome starting at zero and based on the UMD3.1 assembly.

^2^Posterior probability of inclusion (the proportion of MCMC samples in which SNP within the window had non-zero additive genetic variance).

^3^SNP with the highest posterior probability of inclusion within the window.

^4^The B alleles from the Illumina A/B calling system.
