# Supplementary material for: Large-effect pleiotropic or closely linked QTL segregate within and across ten US cattle breeds
Source: BMC Genomics. 2014 Jun 6;15(1):442. doi: 10.1186/1471-2164-15-442 (PMC4102727; doi:10.1186/1471-2164-15-442)
Supplement: Supplementary file 4 — Additional file 4: Large-effect QTL associated with carcass weight in 9 cattle breeds. (DOCX 40 KB) [file 12864_2014_6256_MOESM4_ESM.docx]

**Table S4.** **Large-effect QTL associated with carcass weight in 9 cattle breeds.**

| BTA_Mb^1^ | Start SNP | End SNP | No. SNP | Breed | %V_A_ | PPI^2^ | Lead SNP^3^ | Position (bp) | SNP Effect^4^ | Frequency^4^ |
| --- | --- | --- | --- | --- | --- | --- | --- | --- | --- | --- |
| 6_33 | *rs81127754* | *rs109867538* | 23 | Limousin | 1.01 | 0.66 | *rs29018333* | 33,396,376 | + | 0.81 |
| 6_37 | *rs81128429* | *rs41577868* | 27 | Simmental | 2.27 | 0.90 | *rs81127618* | 37,742,740 | - | 0.10 |
| 6_38 | *rs29010895* | *rs110834363* | 24 | Gelbvieh | 3.19 | 0.83 | *rs81147999* | 38,576,012 | - | 0.28 |
|  |  |  |  | Red Angus | 26.29 | 1.00 | *rs110834363* | 38,939,012 | + | 0.47 |
|  |  |  |  | Simmental | 26.64 | 1.00 | *rs81131480* | 38,869,785 | + | 0.59 |
| 6_39 | *rs81139192* | *rs81129153* | 27 | Simmental | 16.22 | 1.00 | *rs29026121* | 39,216,868 | + | 0.21 |
| 6_54 | *rs109421050* | *rs43465018* | 22 | Limousin | 2.48 | 0.93 | *rs110955860* | 54,324,534 | - | 0.34 |
| 7_93 | *rs109819349* | *rs29009626* | 11 | Angus | 5.62 | 1.00 | *rs110059753* | 93,218,452 | - | 0.29 |
|  |  |  |  | Red Angus | 2.31 | 0.98 | *rs41625563* | 93,073,890 | - | 0.24 |
|  |  |  |  | Simmental | 3.56 | 1.00 | *rs110059753* | 93,218,452 | - | 0.64 |
| 12_32 | *rs110250663* | *rs41580152* | 16 | Red Angus | 1.09 | 0.68 | *rs41623385* | 32,840,998 | + | 0.41 |
| 12_48 | *rs41568048* | *rs110268406* | 26 | Angus | 1.05 | 0.71 | *rs29025358* | 48,455,510 | + | 0.19 |
| 14_3 | *rs109545018* | *rs81157343* | 22 | Shorthorn | 4.09 | 0.81 | *rs110461662* | 3,765,019 | - | 0.72 |
| 14_23 | *rs41724672* | *rs81176130* | 20 | Simmental | 3.05 | 0.91 | *rs41628383* | 23,853,811 | - | 0.68 |
| 14_24 | *rs110845339* | *rs41627956* | 17 | Gelbvieh | 3.63 | 0.85 | *rs42649775* | 24,437,778 | - | 0.30 |
|  |  |  |  | Simmental | 2.58 | 0.96 | *rs41724332* | 24,643,266 | - | 0.23 |
| 14_25 | *rs41627954* | *rs42298470* | 21 | Gelbvieh | 1.48 | 0.57 | *rs29021334* | 25,612,510 | - | 0.38 |
|  |  |  |  | Simmental | 3.22 | 0.90 | *rs110774011* | 25,698,286 | + | 0.38 |
| 14_26 | *rs81143942* | *rs81157855* | 25 | Brangus | 1.54 | 0.64 | *rs81118326* | 26,473,490 | - | 0.27 |
|  |  |  |  | Simmental | 1.28 | 0.75 | *rs41627962* | 26,542,736 | - | 0.68 |
| 16_50 | *rs29018089* | *rs110161051* | 27 | Brangus | 1.41 | 0.56 | *rs110026174* | 50,862,929 | + | 0.48 |
| 20_4 | *rs109377243* | *rs43094958* | 28 | Angus | 3.17 | 0.99 | *rs43350564* | 4,618,689 | + | 0.46 |
|  |  |  |  | Red Angus | 6.55 | 1.00 | *rs43350564* | 4,618,689 | + | 0.39 |
|  |  |  |  | Simmental | 3.98 | 1.00 | *rs43350564* | 4,618,689 | + | 0.21 |
| 20_5 | *rs110348071* | *rs29020081* | 29 | Shorthorn | 1.87 | 0.61 | *rs110348071* | 5,015,135 | + | 0.54 |
| 29_16 | *rs41589158* | *rs41649465* | 22 | Charolais | 4.24 | 0.26 | *rs110871642* | 16,414,885 | - | 0.55 |
| 29_30 | *rs110651226* | *rs109575701* | 24 | Maine-Anjou | 3.28 | 0.71 | *rs41651735* | 30,691,750 | + | 0.69 |

^1^Bovine chromosome and n^th^ 1 Mb window on the same chromosome starting at zero and based on the UMD3.1 assembly.

^2^Posterior probability of inclusion (the proportion of MCMC samples in which SNP within the window had non-zero additive genetic variance).

^3^SNP with the highest posterior probability of inclusion within the window.

^4^The B alleles from the Illumina A/B calling system.
