# Supplementary material for: Large-effect pleiotropic or closely linked QTL segregate within and across ten US cattle breeds
Source: BMC Genomics. 2014 Jun 6;15(1):442. doi: 10.1186/1471-2164-15-442 (PMC4102727; doi:10.1186/1471-2164-15-442)
Supplement: Supplementary file 5 — Additional file 5: Large-effect QTL associated with fat thickness in 9 cattle breeds. (DOCX 35 KB) [file 12864_2014_6256_MOESM5_ESM.docx]

**Table S5.** **Large-effect QTL associated with fat thickness in 9 cattle breeds.**

| BTA_Mb^1^ | Start SNP | End SNP | No. SNP | Breed | %V_A_ | PPI^2^ | Lead SNP^3^ | Position (bp) | SNP Effect^4^ | Frequency^4^ |
| --- | --- | --- | --- | --- | --- | --- | --- | --- | --- | --- |
| 1_78 | *rs29009958* | *rs110348226* | 16 | Hereford | 1.13 | 0.73 | *rs109034747* | 78,088,393 | - | 0.41 |
| 1_141 | *rs41623315* | *rs29024836* | 19 | Brangus | 1.19 | 0.28 | *rs109968527* | 141,807,977 | + | 0.24 |
| 6_33 | *rs81127754* | *rs109867538* | 23 | Angus | 1.47 | 0.81 | *rs81127797* | 33,026,144 | - | 0.06 |
| 6_38 | *rs29010895* | *rs110834363* | 24 | Red Angus | 3.96 | 0.98 | *rs110834363* | 38,939,012 | - | 0.47 |
| 7_94 | *rs41574715* | *rs41656975* | 16 | Hereford | 2.37 | 0.99 | *rs29017030* | 94,573,436 | - | 0.55 |
| 7_96 | *rs81160457* | *rs109967460* | 14 | Simmental | 1.04 | 0.84 | *rs110042721* | 96,844,966 | - | 0.76 |
| 13_68 | *rs41632267* | *rs29012197* | 15 | Hereford | 1.05 | 0.79 | *rs108963848* | 68,133,617 | - | 0.63 |
| 14_75 | *rs109527503* | *rs43747662* | 18 | Angus | 1.03 | 0.68 | *rs110340461* | 75,964,148 | + | 0.57 |
| 16_51 | *rs110196358* | *rs41812411* | 31 | Simmental | 1.08 | 0.94 | *rs41665565* | 51,436,142 | - | 0.27 |
| 17_62 | *rs109709179* | *rs109397161* | 25 | Hereford | 2.61 | 0.93 | *rs109991451* | 62,662,034 | + | 0.57 |
| 20_4 | *rs109377243* | *rs43094958* | 28 | Hereford | 3.98 | 1.00 | *rs43350564* | 4,618,689 | - | 0.56 |
| 21_11 | *rs109746936* | *rs29009998* | 18 | Charolais | 1.28 | 0.14 | *rs29010172* | 11,119,797 | - | 0.31 |
| 25_8 | *rs110665834* | *rs110779607* | 18 | Brangus | 1.12 | 0.35 | *rs29027063* | 8,293,697 | + | 0.59 |
| 26_2 | *rs42847918* | *rs41575907* | 15 | Simmental | 1.24 | 0.97 | *rs110041046* | 2,648,036 | + | 0.80 |

^1^Bovine chromosome and n^th^ 1 Mb window on the same chromosome starting at zero and based on the UMD3.1 assembly.

^2^Posterior probability of inclusion (the proportion of MCMC samples in which SNP within the window had non-zero additive genetic variance).

^3^SNP with the highest posterior probability of inclusion within the window.

^4^The B alleles from the Illumina A/B calling system.
