# Supplementary material for: Large-effect pleiotropic or closely linked QTL segregate within and across ten US cattle breeds
Source: BMC Genomics. 2014 Jun 6;15(1):442. doi: 10.1186/1471-2164-15-442 (PMC4102727; doi:10.1186/1471-2164-15-442)
Supplement: Supplementary file 6 — Additional file 6: Large-effect QTL associated with marbling in 10 cattle breeds. (DOCX 37 KB) [file 12864_2014_6256_MOESM6_ESM.docx]

**Table S6.** **Large-effect QTL associated with marbling in 10 cattle breeds.**

| BTA_Mb^1^ | Start SNP | End SNP | No. SNP | Breed | %V_A_ | PPI^2^ | Lead SNP^3^ | Position (bp) | SNP Effect^4^ | Frequency^4^ |
| --- | --- | --- | --- | --- | --- | --- | --- | --- | --- | --- |
| 1_48 | *rs110988233* | *rs110340489* | 24 | Angus | 1.34 | 0.78 | *rs41565305* | 48,181,287 | + | 0.19 |
| 2_6 | *rs29010906* | *rs41626743* | 11 | Limousin | 5.37 | 0.97 | *rs41638273* | 6,700,805 | - | 0.79 |
| 4_96 | *rs41590661* | *rs109716141* | 29 | Limousin | 3.21 | 0.90 | *rs41590644* | 96,529,890 | - | 0.81 |
| 5_11 | *rs42450190* | *rs109662885* | 19 | Gelbvieh | 4.42 | 0.45 | *rs29019788* | 11,647,027 | + | 0.12 |
| 5_43 | *rs29014396* | *rs43432834* | 25 | Gelbvieh | 8.43 | 0.69 | *rs43432852* | 43,940,765 | + | 0.33 |
| 5_49 | *rs41565111* | *rs41664132* | 15 | Brangus | 7.77 | 0.85 | *rs109534404* | 49,650,207 | - | 0.58 |
| 7_20 | *rs41588220* | *rs43503652* | 25 | Brangus | 1.11 | 0.46 | *rs110909046* | 20,662,867 | - | 0.47 |
| 7_21 | *rs43508635* | *rs43501063* | 25 | Brangus | 1.01 | 0.47 | *rs110835938* | 21,595,908 | + | 0.61 |
| 9_16 | *rs43584258* | *rs42727023* | 26 | Hereford | 1.73 | 0.83 | *rs42989034* | 16,801,734 | - | 0.43 |
| 11_87 | *rs109138849* | *rs108977582* | 32 | Brangus | 1.19 | 0.57 | *rs110795341* | 87,405,137 | - | 0.37 |
| 13_8 | *rs109205261* | *rs41680437* | 13 | Limousin | 1.76 | 0.70 | *rs29010167* | 8,831,875 | + | 0.55 |
| 13_72 | *rs109622520* | *rs42510412* | 28 | Gelbvieh | 2.43 | 0.48 | *rs110270042* | 72,280,054 | - | 0.72 |
| 16_46 | *rs110887322* | *rs41808675* | 18 | Gelbvieh | 3.85 | 0.61 | *rs109670817* | 46,093,561 | - | 0.53 |
| 17_5 | *rs42927497* | *rs109541510* | 27 | Brangus | 1.20 | 0.57 | *rs110882628* | 5,357,573 | + | 0.38 |
| 17_15 | *rs43073507* | *rs109009354* | 24 | Shorthorn | 1.77 | 0.58 | *rs41573240* | 15,271,899 | - | 0.88 |
| 18_46 | *rs110467149* | *rs109861678* | 29 | Red Angus | 1.08 | 0.55 | *rs41884907* | 46,063,097 | - | 0.33 |
| 23_48 | *rs110729869* | *rs29009806* | 30 | Gelbvieh | 1.55 | 0.48 | *rs110646134* | 48,253,839 | - | 0.46 |
| 24_37 | *rs43084616* | *rs42053206* | 27 | Red Angus | 1.58 | 0.69 | *rs110613728* | 37,819,368 | - | 0.57 |
| 24_39 | *rs41569898* | *rs110646611* | 20 | Gelbvieh | 10.49 | 0.89 | *rs109482093* | 39,800,838 | - | 0.91 |
| 25_41 | *rs81126386* | *rs109194521* | 27 | Red Angus | 1.29 | 0.54 | *rs109847518* | 41,213,862 | - | 0.60 |

^1^Bovine chromosome and n^th^ 1 Mb window on the same chromosome starting at zero and based on the UMD3.1 assembly.

^2^Posterior probability of inclusion (the proportion of MCMC samples in which SNP within the window had non-zero additive genetic variance).

^3^SNP with the highest posterior probability of inclusion within the window.

^4^The B alleles from the Illumina A/B calling system.
