# Supplementary material for: Large-effect pleiotropic or closely linked QTL segregate within and across ten US cattle breeds
Source: BMC Genomics. 2014 Jun 6;15(1):442. doi: 10.1186/1471-2164-15-442 (PMC4102727; doi:10.1186/1471-2164-15-442)
Supplement: Supplementary file 7 — Additional file 7: Large-effect QTL associated with mature weight in 2 cattle breeds. (DOCX 33 KB) [file 12864_2014_6256_MOESM7_ESM.docx]

**Table S7.** **Large-effect QTL associated with mature weight in 2 cattle breeds.**

| BTA_Mb^1^ | Start SNP | End SNP | No. SNP | Breed | %V_A_ | PPI^2^ | Lead SNP^3^ | Position (bp) | SNP Effect^4^ | Frequency^4^ |
| --- | --- | --- | --- | --- | --- | --- | --- | --- | --- | --- |
| 1_76 | *rs110041762* | *rs110789800* | 17 | Hereford | 2.19 | 0.98 | *rs110564947* | 76,416,854 | + | 0.28 |
| 3_53 | *rs110142098* | *rs110397079* | 24 | Hereford | 1.32 | 0.96 | *rs110142098* | 53,000,242 | + | 0.28 |
| 3_72 | *rs42404786* | *rs43342803* | 12 | Hereford | 1.23 | 0.96 | *rs110505759* | 72,519,744 | - | 0.84 |
| 5_106 | *rs109969273* | *rs110912524* | 20 | Hereford | 3.90 | 1.00 | *rs110421124* | 106,269,362 | - | 0.38 |
| 6_38 | *rs29010895* | *rs110834363* | 24 | Hereford | 13.57 | 1.00 | *rs81131471* | 38,914,175 | + | 0.93 |
| 7_21 | *rs43508635* | *rs43501063* | 25 | Hereford | 1.21 | 0.94 | *rs110835938* | 21,595,908 | + | 0.29 |
| 7_93 | *rs109819349* | *rs29009626* | 11 | Hereford | 2.84 | 1.00 | *rs110059753* | 93,218,452 | - | 0.46 |
| 15_38 | *rs109164374* | *rs109550701* | 18 | Hereford | 1.18 | 0.96 | *rs110258802* | 38,178,809 | + | 0.24 |
| 20_4 | *rs109377243* | *rs43094958* | 28 | Angus | 2.73 | 0.96 | *rs43350564* | 4,618,689 | + | 0.44 |
|  |  |  |  | Hereford | 18.38 | 1.00 | *rs43349755* | 4,746,836 | + | 0.52 |

^1^Bovine chromosome and n^th^ 1 Mb window on the same chromosome starting at zero and based on the UMD3.1 assembly.

^2^Posterior probability of inclusion (the proportion of MCMC samples in which SNP within the window had non-zero additive genetic variance).

^3^SNP with the highest posterior probability of inclusion within the window.

^4^The B alleles from the Illumina A/B calling system.
