# Supplementary material for: Large-effect pleiotropic or closely linked QTL segregate within and across ten US cattle breeds
Source: BMC Genomics. 2014 Jun 6;15(1):442. doi: 10.1186/1471-2164-15-442 (PMC4102727; doi:10.1186/1471-2164-15-442)
Supplement: Supplementary file 8 — Additional file 8: Large-effect QTL associated with ribeye muscle area in 10 cattle breeds. (DOCX 39 KB) [file 12864_2014_6256_MOESM8_ESM.docx]

**Table S8.** **Large-effect QTL associated with ribeye muscle area in 10 cattle breeds.**

| BTA_Mb^1^ | Start SNP | End SNP | No. SNP | Breed | %V_A_ | PPI^2^ | Lead SNP^3^ | Position (bp) | SNP Effect^4^ | Frequency^4^ |
| --- | --- | --- | --- | --- | --- | --- | --- | --- | --- | --- |
| 1_60 | *rs43241979* | *rs109000991* | 26 | Angus | 1.05 | 0.91 | *rs43710703* | 60,140,981 | - | 0.78 |
| 2_6 | *rs29010906* | *rs41626743* | 11 | Limousin | 11.81 | 1.00 | *rs110233897* | 6,675,045 | + | 0.86 |
| 2_38 | *rs41626579* | *rs81131819* | 19 | Limousin | 1.85 | 0.90 | *rs41645110* | 38,525,454 | - | 0.80 |
| 3_112 | *rs109954761* | *rs109736826* | 29 | Shorthorn | 5.93 | 0.56 | *rs29016125* | 112,062,155 | - | >0.99 |
| 3_113 | *rs109692471* | *rs42765400* | 22 | Shorthorn | 1.16 | 0.23 | *rs41586343* | 113,606,642 | - | >0.99 |
| 5_34 | *rs110704246* | *rs110336027* | 18 | Shorthorn | 3.87 | 0.38 | *rs110565093* | 34,220,929 | - | >0.99 |
| 5_48 | *rs29016809* | *rs41599228* | 14 | Angus | 1.26 | 0.93 | *rs29027133* | 48,460,111 | + | 0.82 |
|  |  |  |  | Brangus | 1.11 | 0.39 | *rs41657459* | 48,834,486 | + | 0.27 |
| 5_50 | *rs109005290* | *rs41597788* | 15 | Brangus | 1.16 | 0.41 | *rs41565113* | 50,229,041 | - | 0.79 |
| 6_38 | *rs29010895* | *rs110834363* | 24 | Simmental | 1.52 | 0.84 | *rs110834363* | 38,939,012 | + | 0.52 |
| 7_16 | *rs41658749* | *rs109444369* | 24 | Hereford | 1.62 | 0.94 | *rs110086015* | 16,675,279 | - | 0.69 |
| 7_93 | *rs109819349* | *rs29009626* | 11 | Angus | 5.40 | 1.00 | *rs110059753* | 93,218,452 | - | 0.29 |
|  |  |  |  | Gelbvieh | 7.90 | 0.46 | *rs109819349* | 93,007,435 | - | 0.79 |
|  |  |  |  | Hereford | 4.96 | 1.00 | *rs110059753* | 93,218,452 | - | 0.46 |
|  |  |  |  | Simmental | 2.56 | 1.00 | *rs110059753* | 93,218,452 | - | 0.63 |
| 9_49 | *rs43591106* | *rs41662693* | 22 | Charolais | 1.52 | 0.17 | *rs42478960* | 49,597,855 | - | 0.29 |
| 10_21 | *rs41644777* | *rs109743700* | 24 | Angus | 1.37 | 0.93 | *rs110890764* | 21,225,382 | + | 0.27 |
| 11_49 | *rs108980261* | *rs29020516* | 22 | Red Angus | 1.51 | 0.74 | *rs41665730* | 49,473,033 | - | 0.28 |
| 15_38 | *rs109164374* | *rs109550701* | 18 | Hereford | 2.46 | 0.96 | *rs109164374* | 38,003,134 | + | 0.47 |
| 15_82 | *rs110466368* | *rs42781637* | 28 | Shorthorn | 1.36 | 0.50 | *rs43708449* | 82,260,685 | + | 0.28 |
| 16_74 | *rs41824476* | *rs110956939* | 24 | Hereford | 1.07 | 0.77 | *rs109115592* | 74,158,269 | - | 0.43 |
| 20_26 | *rs109921395* | *rs41601571* | 12 | Simmental | 1.22 | 0.90 | *rs81169154* | 26,733,496 | + | 0.71 |
| X_7 | *rs110876560* | *rs110406349* | 11 | Limousin | 1.14 | 0.86 | *rs109517818* | 7,200,715 | + | 0.53 |
| X_25 | *rs81158862* | *rs41580627* | 7 | Gelbvieh | 2.23 | 0.34 | *rs110953081* | 25,731,651 | - | 0.51 |

^1^Bovine chromosome and n^th^ 1 Mb window on the same chromosome starting at zero and based on the UMD3.1 assembly.

^2^Posterior probability of inclusion (the proportion of MCMC samples in which SNP within the window had non-zero additive genetic variance).

^3^SNP with the highest posterior probability of inclusion within the window.

^4^The B alleles from the Illumina A/B calling system.
