# Supplementary material for: Large-effect pleiotropic or closely linked QTL segregate within and across ten US cattle breeds
Source: BMC Genomics. 2014 Jun 6;15(1):442. doi: 10.1186/1471-2164-15-442 (PMC4102727; doi:10.1186/1471-2164-15-442)
Supplement: Supplementary file 9 — Additional file 9: Large-effect QTL associated with weaning weight direct in 10 cattle breeds. (DOCX 43 KB) [file 12864_2014_6256_MOESM9_ESM.docx]

**Table S9.** **Large-effect QTL associated with weaning weight direct in 10 cattle breeds.**

| BTA_Mb^1^ | Start SNP | End SNP | No. SNP | Breed | %V_A_ | PPI^2^ | Lead SNP^3^ | Position (bp) | SNP Effect^4^ | Frequency^4^ |
| --- | --- | --- | --- | --- | --- | --- | --- | --- | --- | --- |
| 2_6 | *rs29010906* | *rs41626743* | 11 | Limousin | 1.37 | 0.63 | *rs110233897* | 6,675,045 | + | 0.87 |
| 3_55 | *rs108949614* | *rs81172555* | 10 | Maine-Anjou | 1.09 | 0.38 | *rs81111696* | 55,537,894 | + | 0.56 |
| 4_61 | *rs109672663* | *rs43399326* | 29 | Maine-Anjou | 1.38 | 0.66 | *rs43400956* | 61,729,628 | - | 0.81 |
| 5_106 | *rs109969273* | *rs110912524* | 20 | Hereford | 1.98 | 0.97 | *rs41654528* | 106,230,591 | - | 0.36 |
| 6_14 | *rs81149576* | *rs81160196* | 26 | Charolais | 1.36 | 0.13 | *rs41658357* | 14,911,170 | - | 0.36 |
| 6_37 | *rs81128429* | *rs41577868* | 27 | Simmental | 1.43 | 0.82 | *rs110737114* | 37,801,349 | + | 0.91 |
| 6_38 | *rs29010895* | *rs110834363* | 24 | Gelbvieh | 1.84 | 0.74 | *rs81128660* | 38,464,203 | + | 0.82 |
|  |  |  |  | Hereford | 7.20 | 1.00 | *rs110834363* | 38,939,012 | + | 0.20 |
|  |  |  |  | Limousin | 13.26 | 1.00 | *rs81131471* | 38,914,175 | + | 0.78 |
|  |  |  |  | Red Angus | 11.39 | 1.00 | *rs110834363* | 38,939,012 | + | 0.47 |
|  |  |  |  | Simmental | 15.80 | 1.00 | *rs81131480* | 38,869,785 | + | 0.59 |
| 6_39 | *rs81139192* | *rs81129153* | 27 | Simmental | 9.60 | 0.99 | *rs110411130* | 39,313,672 | - | 0.08 |
| 6_41 | *rs43463315* | *rs41651246* | 31 | Shorthorn | 1.47 | 0.49 | *rs43459713* | 41,795,944 | + | 0.70 |
| 6_53 | *rs29011427* | *rs41597159* | 25 | Gelbvieh | 1.20 | 0.61 | *rs81131430* | 53,893,718 | - | 0.23 |
| 7_93 | *rs109819349* | *rs29009626* | 11 | Angus | 1.23 | 0.99 | *rs110059753* | 93,218,452 | - | 0.30 |
|  |  |  |  | Brangus | 1.08 | 0.42 | *rs110059753* | 93,218,452 | - | 0.60 |
|  |  |  |  | Hereford | 2.22 | 1.00 | *rs110059753* | 93,218,452 | - | 0.46 |
|  |  |  |  | Red Angus | 1.52 | 0.92 | *rs110059753* | 93,218,452 | - | 0.28 |
|  |  |  |  | Simmental | 2.49 | 1.00 | *rs110059753* | 93,218,452 | - | 0.64 |
| 10_98 | *rs109069721* | *rs42814092* | 27 | Hereford | 1.20 | 0.98 | *rs109650587* | 98,223,275 | + | 0.15 |
| 11_11 | *rs41619289* | *rs42758993* | 26 | Gelbvieh | 1.05 | 0.61 | *rs43665969* | 11,404,878 | + | 0.83 |
| 14_24 | *rs110845339* | *rs41627956* | 17 | Gelbvieh | 2.58 | 0.80 | *rs42649775* | 24,437,778 | - | 0.30 |
|  |  |  |  | Simmental | 3.48 | 0.96 | *rs110383563* | 24,326,513 | + | 0.31 |
| 14_26 | *rs81143942* | *rs81157855* | 25 | Brangus | 1.41 | 0.61 | *rs81118182* | 26,713,734 | - | 0.24 |
|  |  |  |  | Simmental | 1.30 | 0.84 | *rs41627962* | 26,542,736 | - | 0.68 |
| 15_9 | *rs41749553* | *rs42633485* | 18 | Charolais | 2.81 | 0.20 | *rs29023253* | 9,125,948 | + | 0.37 |
| 17_6 | *rs41577199* | *rs110693564* | 25 | Limousin | 1.09 | 0.77 | *rs41835858* | 6,243,273 | - | 0.46 |
| 20_4 | *rs109377243* | *rs43094958* | 28 | Angus | 1.88 | 1.00 | *rs43350564* | 4,618,689 | + | 0.45 |
|  |  |  |  | Hereford | 6.31 | 1.00 | *rs43350564* | 4,618,689 | + | 0.56 |
|  |  |  |  | Red Angus | 3.71 | 1.00 | *rs43350564* | 4,618,689 | + | 0.39 |
|  |  |  |  | Simmental | 2.20 | 0.99 | *rs43350564* | 4,618,689 | + | 0.21 |
| 26_32 | *rs110858406* | *rs42102064* | 26 | Red Angus | 1.53 | 0.86 | *rs109741976* | 32,892,811 | + | 0.17 |
| 29_30 | *rs110651226* | *rs109575701* | 24 | Maine-Anjou | 1.74 | 0.56 | *rs41651735* | 30,691,750 | + | 0.69 |
| X_145 | *rs41565565* | *rs81177875* | 24 | Brangus | 1.71 | 0.59 | *rs110339726* | 145,303,466 | - | 0.60 |

^1^Bovine chromosome and n^th^ 1 Mb window on the same chromosome starting at zero and based on the UMD3.1 assembly.

^2^Posterior probability of inclusion (the proportion of MCMC samples in which SNP within the window had non-zero additive genetic variance).

^3^SNP with the highest posterior probability of inclusion within the window.

^4^The B alleles from the Illumina A/B calling system.
