# Supplementary material for: Large-effect pleiotropic or closely linked QTL segregate within and across ten US cattle breeds
Source: BMC Genomics. 2014 Jun 6;15(1):442. doi: 10.1186/1471-2164-15-442 (PMC4102727; doi:10.1186/1471-2164-15-442)
Supplement: Supplementary file 10 — Additional file 10: Large-effect QTL associated with weaning weight maternal in 10 cattle breeds. (DOCX 41 KB) [file 12864_2014_6256_MOESM10_ESM.docx]

**Table S10.** **Large-effect QTL associated with weaning weight maternal in 10 cattle breeds.**

| BTA_Mb^1^ | Start SNP | End SNP | No. SNP | Breed | %V_A_ | PPI^2^ | Lead SNP^3^ | Position (bp) | SNP Effect^4^ | Frequency^4^ |
| --- | --- | --- | --- | --- | --- | --- | --- | --- | --- | --- |
| 1_2 | *rs110875985* | *rs43212498* | 24 | Charolais | 1.34 | 0.29 | *rs109378326* | 2,524,748 | + | 0.59 |
|  |  |  |  | Hereford | 3.03 | 0.96 | *rs109205247* | 2,462,297 | + | 0.81 |
|  |  |  |  | Simmental | 1.00 | 0.92 | *rs109378326* | 2,524,748 | + | 0.65 |
| 1_10 | *rs29022825* | *rs41621754* | 15 | Maine-Anjou | 2.02 | 0.61 | *rs41619783* | 10,643,238 | + | 0.58 |
| 2_6 | *rs29010906* | *rs41626743* | 11 | Limousin | 5.30 | 0.82 | *rs41638273* | 6,700,805 | - | 0.80 |
| 3_10 | *rs109754043* | *rs41595611* | 21 | Red Angus | 1.19 | 0.65 | *rs109754043* | 10,000,411 | - | 0.29 |
| 3_19 | *rs110186712* | *rs41256309* | 17 | Angus | 1.40 | 0.90 | *rs43709214* | 19,193,451 | + | 0.46 |
| 4_73 | *rs42709546* | *rs43072408* | 23 | Charolais | 2.17 | 0.32 | *rs43403587* | 73,699,663 | - | 0.36 |
| 6_37 | *rs81128429* | *rs41577868* | 27 | Red Angus | 3.74 | 0.97 | *rs81128530* | 37,868,743 | - | 0.53 |
| 9_15 | *rs43581894* | *rs29027235* | 28 | Hereford | 1.06 | 0.88 | *rs43581894* | 15,029,445 | + | 0.49 |
| 11_81 | *rs109655141* | *rs109847109* | 16 | Maine-Anjou | 1.62 | 0.47 | *rs41617764* | 81,484,369 | - | 0.64 |
| 12_54 | *rs43705355* | *rs110361717* | 27 | Brangus | 3.14 | 0.65 | *rs41575415* | 54,111,801 | - | 0.19 |
| 14_24 | *rs110845339* | *rs41627956* | 17 | Simmental | 1.39 | 0.77 | *rs110383563* | 24,326,513 | - | 0.31 |
| 14_50 | *rs108945635* | *rs42379066* | 21 | Simmental | 1.11 | 0.94 | *rs43706062* | 50,784,282 | - | 0.55 |
| 15_17 | *rs29021949* | *rs110525571* | 18 | Maine-Anjou | 1.23 | 0.49 | *rs42424601* | 17,080,803 | - | 0.58 |
| 15_29 | *rs29012314* | *rs110758902* | 27 | Red Angus | 1.64 | 0.84 | *rs43711854* | 29,477,369 | - | 0.74 |
| 17_63 | *rs110964597* | *rs41599571* | 26 | Hereford | 1.49 | 0.81 | *rs110358424* | 63,063,122 | + | 0.37 |
| 18_1 | *rs110131781* | *rs110491665* | 22 | Shorthorn | 1.54 | 0.54 | *rs41603540* | 1,422,084 | - | 0.44 |
| 18_23 | *rs41580584* | *rs81128590* | 28 | Gelbvieh | 1.23 | 0.74 | *rs110672596* | 23,363,011 | - | 0.44 |
| 19_51 | *rs41923412* | *rs109147235* | 27 | Hereford | 1.02 | 0.88 | *rs110446126* | 51,680,150 | + | 0.28 |
| 21_27 | *rs41970372* | *rs42618987* | 22 | Maine-Anjou | 1.36 | 0.52 | *rs29023496* | 27,087,855 | + | 0.47 |
| 22_31 | *rs109903761* | *rs81136706* | 20 | Hereford | 1.35 | 0.93 | *rs29018735* | 31,702,243 | - | 0.38 |
| 24_46 | *rs110563969* | *rs81139085* | 23 | Hereford | 1.04 | 0.85 | *rs110068809* | 46,777,003 | - | 0.50 |
| 25_16 | *rs41586495* | *rs110097742* | 22 | Simmental | 1.14 | 0.94 | *rs41646570* | 16,256,587 | - | 0.40 |
| 25_35 | *rs109405213* | *rs108985965* | 28 | Simmental | 1.49 | 0.99 | *rs109525092* | 35,392,866 | - | 0.66 |
| 29_37 | *rs41651830* | *rs110942558* | 25 | Limousin | 1.14 | 0.42 | *rs29017738* | 37,380,092 | + | 0.68 |
| 29_44 | *rs110552089* | *rs109977592* | 33 | Angus | 1.60 | 0.95 | *rs43709648* | 44,416,282 | - | 0.08 |
| X_140 | *rs42210240* | *rs110640411* | 8 | Maine-Anjou | 1.22 | 0.46 | *rs41627332* | 140,767,673 | - | 0.62 |

^1^Bovine chromosome and n^th^ 1 Mb window on the same chromosome starting at zero and based on the UMD3.1 assembly.

^2^Posterior probability of inclusion (the proportion of MCMC samples in which SNP within the window had non-zero additive genetic variance).

^3^SNP with the highest posterior probability of inclusion within the window.

^4^The B alleles from the Illumina A/B calling system.
