# Supplementary material for: Large-effect pleiotropic or closely linked QTL segregate within and across ten US cattle breeds
Source: BMC Genomics. 2014 Jun 6;15(1):442. doi: 10.1186/1471-2164-15-442 (PMC4102727; doi:10.1186/1471-2164-15-442)
Supplement: Supplementary file 11 — Additional file 11: Large-effect QTL associated with yield grade in 5 cattle breeds. (DOCX 33 KB) [file 12864_2014_6256_MOESM11_ESM.docx]

**Table S11.** **Large-effect QTL associated with yield grade in 5 cattle breeds.**

| BTA_Mb^1^ | Start SNP | End SNP | No. SNP | Breed | %V_A_ | PPI^2^ | Lead SNP^3^ | Position (bp) | SNP Effect^4^ | Frequency^4^ |
| --- | --- | --- | --- | --- | --- | --- | --- | --- | --- | --- |
| 2_3 | *rs110161155* | *rs108939474* | 20 | Red Angus | 2.45 | 0.74 | *rs110161155* | 3,046,092 | - | 0.85 |
| 2_6 | *rs29010906* | *rs41626743* | 11 | Limousin | 13.80 | 1.00 | *rs110233897* | 6,675,045 | - | 0.86 |
| 8_65 | *rs81172265* | *rs109883188* | 21 | Simmental | 1.68 | 0.94 | *rs110309078* | 65,721,612 | - | 0.60 |
| 13_57 | *rs109502430* | *rs41694639* | 22 | Gelbvieh | 1.06 | 0.39 | *rs109257146* | 57,051,358 | - | 0.80 |
| 17_5 | *rs42927497* | *rs109541510* | 27 | Simmental | 1.05 | 0.83 | *rs110882628* | 5,357,573 | + | 0.57 |
| 18_18 | *rs110528295* | *rs110871891* | 26 | Red Angus | 2.25 | 0.79 | *rs110808323* | 18,267,940 | - | 0.50 |
| 19_17 | *rs109495655* | *rs41584880* | 24 | Red Angus | 1.02 | 0.58 | *rs109576264* | 17,189,453 | - | 0.73 |
| 20_4 | *rs109377243* | *rs43094958* | 28 | Red Angus | 2.04 | 0.80 | *rs43349755* | 4,746,836 | + | 0.56 |
| 20_5 | *rs110348071* | *rs29020081* | 29 | Simmental | 1.12 | 0.86 | *rs110323614* | 5,392,124 | - | 0.41 |
| 26_42 | *rs41604406* | *rs109153374* | 24 | Red Angus | 3.93 | 0.95 | *rs110802361* | 42,471,130 | + | 0.59 |

^1^Bovine chromosome and n^th^ 1 Mb window on the same chromosome starting at zero and based on the UMD3.1 assembly.

^2^Posterior probability of inclusion (the proportion of MCMC samples in which SNP within the window had non-zero additive genetic variance).

^3^SNP with the highest posterior probability of inclusion within the window.

^4^The B alleles from the Illumina A/B calling system.
