# Supplementary material for: Large-effect pleiotropic or closely linked QTL segregate within and across ten US cattle breeds
Source: BMC Genomics. 2014 Jun 6;15(1):442. doi: 10.1186/1471-2164-15-442 (PMC4102727; doi:10.1186/1471-2164-15-442)
Supplement: Supplementary file 12 — Additional file 12: Large-effect QTL associated with yearling weight in 10 cattle breeds. (DOCX 42 KB) [file 12864_2014_6256_MOESM12_ESM.docx]

**Table S12.** **Large-effect QTL associated with yearling weight in 10 cattle breeds.**

| BTA_Mb^1^ | Start SNP | End SNP | No. SNP | Breed | %V_A_ | PPI^2^ | Lead SNP^3^ | Position (bp) | SNP Effect^4^ | Frequency^4^ |
| --- | --- | --- | --- | --- | --- | --- | --- | --- | --- | --- |
| 3_41 | *rs29013292* | *rs110586379* | 18 | Charolais | 1.18 | 0.10 | *rs41617640* | 41,288,830 | - | 0.43 |
| 4_61 | *rs109672663* | *rs43399326* | 29 | Maine-Anjou | 1.53 | 0.67 | *rs43400956* | 61,729,628 | - | 0.81 |
| 5_106 | *rs109969273* | *rs110912524* | 20 | Hereford | 4.89 | 1.00 | *rs41654528* | 106,230,591 | - | 0.36 |
| 6_37 | *rs81128429* | *rs41577868* | 27 | Simmental | 1.57 | 0.92 | *rs81127618* | 37,742,740 | - | 0.10 |
| 6_38 | *rs29010895* | *rs110834363* | 24 | Hereford | 9.99 | 1.00 | *rs81131471* | 38,914,175 | + | 0.93 |
|  |  |  |  | Limousin | 22.97 | 1.00 | *rs81131471* | 38,914,175 | + | 0.78 |
|  |  |  |  | Red Angus | 12.51 | 1.00 | *rs110834363* | 38,939,012 | + | 0.47 |
|  |  |  |  | Simmental | 15.13 | 1.00 | *rs81131480* | 38,869,785 | + | 0.59 |
| 6_39 | *rs81139192* | *rs81129153* | 27 | Simmental | 7.65 | 0.99 | *rs110411130* | 39,313,672 | - | 0.08 |
| 6_41 | *rs43463315* | *rs41651246* | 31 | Shorthorn | 2.54 | 0.57 | *rs43459713* | 41,795,944 | + | 0.70 |
| 6_44 | *rs43465435* | *rs81130717* | 27 | Maine-Anjou | 1.06 | 0.50 | *rs81130510* | 44,926,243 | + | 0.72 |
| 6_54 | *rs109421050* | *rs43465018* | 22 | Gelbvieh | 1.51 | 0.65 | *rs110955860* | 54,324,534 | - | 0.18 |
| 7_6 | *rs41657401* | *rs110159875* | 23 | Hereford | 1.13 | 0.82 | *rs41657401* | 6,010,779 | - | 0.55 |
| 7_85 | *rs110892056* | *rs41670188* | 24 | Shorthorn | 1.05 | 0.48 | *rs42998760* | 85,579,651 | - | 0.31 |
| 7_93 | *rs109819349* | *rs29009626* | 11 | Angus | 1.27 | 1.00 | *rs110059753* | 93,218,452 | - | 0.30 |
|  |  |  |  | Hereford | 2.70 | 0.99 | *rs110059753* | 93,218,452 | - | 0.46 |
|  |  |  |  | Red Angus | 1.52 | 0.94 | *rs41625563* | 93,073,890 | - | 0.24 |
|  |  |  |  | Simmental | 2.78 | 1.00 | *rs110059753* | 93,218,452 | - | 0.64 |
| 14_23 | *rs41724672* | *rs81176130* | 20 | Simmental | 1.68 | 0.81 | *rs41628383* | 23,853,811 | - | 0.68 |
| 14_24 | *rs110845339* | *rs41627956* | 17 | Gelbvieh | 1.67 | 0.74 | *rs42649775* | 24,437,778 | - | 0.30 |
|  |  |  |  | Simmental | 2.72 | 0.97 | *rs110383563* | 24,326,513 | + | 0.31 |
| 14_25 | *rs41627954* | *rs42298470* | 21 | Simmental | 1.87 | 0.80 | *rs110774011* | 25,698,286 | + | 0.38 |
| 14_26 | *rs81143942* | *rs81157855* | 25 | Brangus | 1.25 | 0.58 | *rs81118326* | 26,473,490 | - | 0.27 |
| 15_39 | *rs109499279* | *rs81124308* | 17 | Hereford | 1.56 | 0.84 | *rs41630328* | 39,096,970 | - | 0.52 |
| 19_63 | *rs42700237* | *rs109935872* | 22 | Charolais | 1.01 | 0.11 | *rs41923722* | 63,261,405 | + | 0.57 |
| 20_4 | *rs109377243* | *rs43094958* | 28 | Angus | 2.57 | 1.00 | *rs41931625* | 4,496,376 | - | 0.21 |
|  |  |  |  | Hereford | 7.05 | 1.00 | *rs43349755* | 4,746,836 | + | 0.52 |
|  |  |  |  | Red Angus | 4.19 | 1.00 | *rs43350564* | 4,618,689 | + | 0.39 |
|  |  |  |  | Simmental | 2.72 | 1.00 | *rs43350564* | 4,618,689 | + | 0.21 |
| 26_16 | *rs42736384* | *rs110543541* | 20 | Charolais | 1.14 | 0.11 | *rs42083758* | 16,916,860 | + | 0.77 |
| 29_30 | *rs110651226* | *rs109575701* | 24 | Maine-Anjou | 3.79 | 0.82 | *rs41651735* | 30,691,750 | + | 0.69 |

^1^Bovine chromosome and n^th^ 1 Mb window on the same chromosome starting at zero and based on the UMD3.1 assembly.

^2^Posterior probability of inclusion (the proportion of MCMC samples in which SNP within the window had non-zero additive genetic variance).

^3^SNP with the highest posterior probability of inclusion within the window.

^4^The B alleles from the Illumina A/B calling system.
